# Using Bayesian Multilevel Whole Genome Regression Models for Partial Pooling of Training Sets in Genomic Prediction

**DOI:** 10.1534/g3.115.019299

**Published:** 2015-05-29

**Authors:** Frank Technow, L. Radu Totir

**Affiliations:** DuPont Pioneer, Johnston, Iowa 50131

**Keywords:** genomic selection, GenPred, shared data resource, genomic prediction, data pooling, Bayesian whole-genome regression, multilevel models, multiple populations

## Abstract

Training set size is an important determinant of genomic prediction accuracy. Plant breeding programs are characterized by a high degree of structuring, particularly into populations. This hampers the establishment of large training sets for each population. Pooling populations increases training set size but ignores unique genetic characteristics of each. A possible solution is partial pooling with multilevel models, which allows estimating population-specific marker effects while still leveraging information across populations. We developed a Bayesian multilevel whole-genome regression model and compared its performance with that of the popular BayesA model applied to each population separately (no pooling) and to the joined data set (complete pooling). As an example, we analyzed a wide array of traits from the nested association mapping maize population. There we show that for small population sizes (*e.g.*, <50), partial pooling increased prediction accuracy over no or complete pooling for populations represented in the training set. No pooling was superior; however, when populations were large. In another example data set of interconnected biparental maize populations either partial or complete pooling was superior, depending on the trait. A simulation showed that no pooling is superior when differences in genetic effects among populations are large and partial pooling when they are intermediate. With small differences, partial and complete pooling achieved equally high accuracy. For prediction of new populations, partial and complete pooling had very similar accuracy in all cases. We conclude that partial pooling with multilevel models can maximize the potential of pooling by making optimal use of information in pooled training sets.

Genomic selection (Meuwissen *et al.* 2001) in animal and plant breeding rests on the accurate prediction of genomic breeding values (GEBVs). An important determinant of prediction accuracy is the size of the training set (Daetwyler *et al.* 2010). In animal breeding, assembling large training sets is relatively straight forward for large dairy breeds like Holstein Friesian, where genomic selection is applied most successfully to date (Hayes *et al.* 2009b). For smaller dairy cattle breeds and in particular for beef cattle breeds, however, assembling sufficiently large training sets within each breed is often not possible (Weber *et al.* 2012). The creation of multipopulation training sets by pooling several breeds is therefore of great interest and subject of current research (De Los Campos and Sorensen 2014; Lund *et al.* 2014).

A similar situation exists in plant breeding, which is characterized by a high degree of structuring (Albrecht *et al.* 2014). This structuring results from the importance of keeping distinct heterotic groups for maximum exploitation of heterosis (Melchinger and Gumber 1998), from the predominance of distinct biparental populations (Mikel and Dudley 2006), and the need for specialized breeding programs targeting specific traits or environments (Windhausen *et al.* 2012). This requires that the phenotyping and genotyping resources available to a breeding program have to be allocated to multiple populations, which prevents the creation of sufficiently large training sets for each population. Several studies therefore investigated the merit of pooled training sets combining populations (Asoro *et al.* 2011; Heffner *et al.* 2011; Lorenz *et al.* 2012; Riedelsheimer *et al.* 2013; Lehermeier *et al.* 2014) or even heterotic groups (Technow *et al.* 2013; Lehermeier *et al.* 2014).

However, pooling training sets is complicated by the unique genetic characteristics of populations, that arise because of differences in linkage disequilibrium or allele frequency, (Weber *et al.* 2012; Windhausen *et al.* 2012; Riedelsheimer *et al.* 2013; Technow *et al.* 2014b), quantitative trait locus (QTL) by background interaction (Blanc *et al.* 2006; Melchinger *et al.* 2008), and presence of population specific QTL alleles (Buckler *et al.* 2009; Giraud *et al.* 2014). This might be the reason why using pooled training sets failed to increase prediction accuracy in some applications in plant (Desta and Ortiz 2014) and animal breeding (Lund *et al.* 2014).

Therefore, Brøndum *et al.* (2012) proposed to use separate training sets for each population but to derive genome position specific priors from estimation results in the other population. In this way, unique genome properties of each population could be accounted for while still using information from other populations. A similar, but perhaps more formal, approach is *partial pooling*, facilitated by Bayesian multilevel models (Gelman and Hill 2006; Gelman and Pardoe 2006; Gelman 2006a). In multilevel models, specific marker effects are estimated for each population. However, the prior means of these specific marker effects, which might be interpreted as overall or unspecific marker effects, are estimated from data of all populations, simultaneously with the specific marker effects. Because the specific marker effects are shrunk toward the overall effects, the former are still informed by data from the other populations to a certain degree. Partial pooling thus strikes a middle ground between *no pooling* (specific marker effects estimated from data of the specific population only) and *complete pooling* (common marker effects estimated from pooled training sets).

Our objectives were to (i) demonstrate the use of Bayesian multilevel whole-genome regression models for genomic prediction and (ii) investigate scenarios in which partial pooling might be superior over no or complete pooling of training sets. Our investigations were based on two publicly available maize breeding data sets and supported by a simulation study.

## Materials and Methods

### Multilevel whole-genome regression model

The model fitted to the data were as follows:

$$\begin{array}{l} y_{ij}∼N\left(\mu_{ij},\sigma_{e}^{2}\right)\\ \mu_{ij}=\beta_{0}+\sum_{k}z_{ijk}u_{jk}, \end{array}$$ (1)

where $y_{ij}$ was the observed phenotypic value of the $i^{th}$ individual from the $j^{th}$ population and $\mu_{ij}$ its linear predictor. The phenotypic data $y_{ij}$ was centered to mean zero and scaled to unit variance. The Normal density function, which was used as likelihood function, was denoted as N with $\sigma_{e}^{2}$ denoting the residual variance component. The common intercept was $\beta_{0}$. Finally, $u_{jk}$ denoted the additive effect of the $k^{th}$ biallelic single-nucleotide polymorphism (SNP) marker in population *j*. The genotype of individual *i* from population *j* at marker *k* was represented by $z_{ijk}$, which was the number of reference alleles, centered by twice the reference allele frequency. Which of the alleles was chosen as reference allele depended on the data set and is described below. Effects $u_{jk}$ were only estimated when the corresponding marker was polymorphic in population *j*. Otherwise it was set to 0 and treated as a constant.

**Figure 1:**
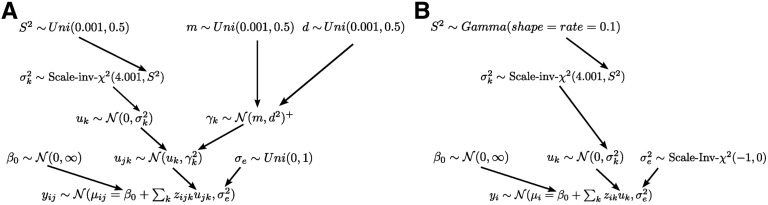
Graphic visualization of the multilevel model (A) and the conventional BayesA model (B).

The hierarchical prior distribution setup will be explained next. A graphical display is shown in Figure 1A. The prior of $u_{jk}$ was

$$u_{jk}∼N\left(u_{k},\gamma_{k}^{2}\right),$$ (2)

where *u_k_* was the overall effect of the $k^{th}$ marker and variance parameter $\gamma_{k}^{2}$ quantified the deviations of the specific effects $u_{jk}$ from $u_{k}$. Note that all else equal, the shrinkage toward $u_{k}$ is the stronger the smaller $\gamma_{k}^{2}$.

Both parameters were associated with prior distributions themselves and estimated from the data. For $u_{k}$ this was $u_{k}∼N\left(0,\sigma_{k}^{2}\right)$. Here, the variance parameter $\sigma_{k}^{2}$ controls the amount of shrinkage toward 0. It was associated with a scaled inverse χ^2^ prior with 4.001 degree of freedom and scale parameter $S^{2}$. The prior for $u_{k}$ thus corresponded to the well-known “BayesA” prior (Meuwissen *et al.* 2001). The prior for the intercept $\beta_{0}$ was a Normal distribution with mean 0 and a very large variance.

For the variance parameter $\gamma_{k}^{2}$, we specified

$$\gamma_{k}∼N{\left(m,d^{2}\right)}^{+}$$ (3)

which is a Normal distribution prior on $\gamma_{k}$ with mean parameter *m* and SD *d*, left truncated at zero. Note that the mean of the truncated distribution $N{\left(m,d^{2}\right)}^{+}$, which is a function of *m*, *d*, and the truncation points, can be interpreted as the “typical” deviation of the specific marker effects $u_{jk}$ from $u_{k}$. Greater values of this mean indicate larger deviations and vice versa. This parameter might therefore be used to quantify population divergence. We chose a truncated Normal as prior distribution because it is straightforward to specify and interpret its hyperparameters and to potentially include prior knowledge. It is also our experience that a truncated Normal prior can be more robust and improve convergence compared to other potential choices such as Gamma distributions.

A Uniform distribution prior Uni(0.001,0.5) was used for the hyperparameters $S^{2}$, *m*, and *d*. For the residual variance $\sigma_{e}^{2}$ we specified a Uniform distribution prior over the interval [0, 1] on $\sigma_{e}$, which agrees with recommendations for uninformative priors on variance components (Gelman, 2006b). We used Uniform distributions because they allow us to convey prior ignorance while still bounding the parameters to a sensible value range. The latter feature can improve robustness and convergence. In contrast to other choices for uninformative priors, such as improper distributions, the implications of Uniform distributions also are readily apparent to researchers less familiar with Bayesian statistics. Gelman (2006b) state further reasons for why a proper Uniform distribution might be preferable over improper uniform distributions. However, we note that our choice of prior distributions differs from those commonly used for Bayesian whole genome regression, which are chosen mainly for their conjugacy and computational efficiency. Although we see several advantages in choosing Uniform and truncated Normal distribution priors, their lack of conjugacy is a drawback and can be associated with computational performance penalties.

Samples from the posterior distribution were drawn with Gibbs sampling, implemented in the JAGS Gibbs sampling environment (Plummer 2003). The total number of samples was 1000, drawn from a single chain with burn in of 10,000 and thinning intervals of 500. These settings ensured convergence and an effective sample size (ESS) of >100 for all parameters (ESS of $u_{k}$ and $u_{jk}$ were typically >500).

The ESS was calculated with the R (R Core Team 2013) package CODA (Plummer *et al.* 2006), which was also used to monitor convergence using diagnostic plots.

### Conventional whole-genome regression model

We used the popular Bayesian whole-genome regression method “BayesA” (Meuwissen *et al.* 2001), with the modifications of Yang and Tempelman (2012) pertaining to the hyperparameter $S^{2}$ (see Figure 1B for a graphical representation). The linear model was

$$\begin{array}{l} y_{i}∼N\left(\mu_{i},\sigma_{e}^{2}\right)\\ \mu_{i}=\beta_{0}+\sum_{k}z_{ik}u_{k}, \end{array}$$ (4)

which is principally the same as in (1), with the difference that the population index *j* was dropped. For no pooling, the model was applied to each population in turn, for complete pooling to the joint data set. For $\sigma_{e}^{2}$ we used an improper scaled inverse χ^2^ prior with −1 degrees of freedom and scale equal to zero. This is equivalent to an improper uniform prior density on $\sigma_{e}$ (Gelman 2006b; Yang and Tempelman 2012), which is similar to the proper Uniform density that was used for the multilevel model but exploits conjugacy.

The BayesA Gibbs Sampler was implemented as a C routine compatible with the R statistical software environment. Again we drew a total number of 1000 samples from a single chain with burn in of 10,000 and thinning of 500.

### Estimation, prediction, and testing procedure

**Figure 2:**
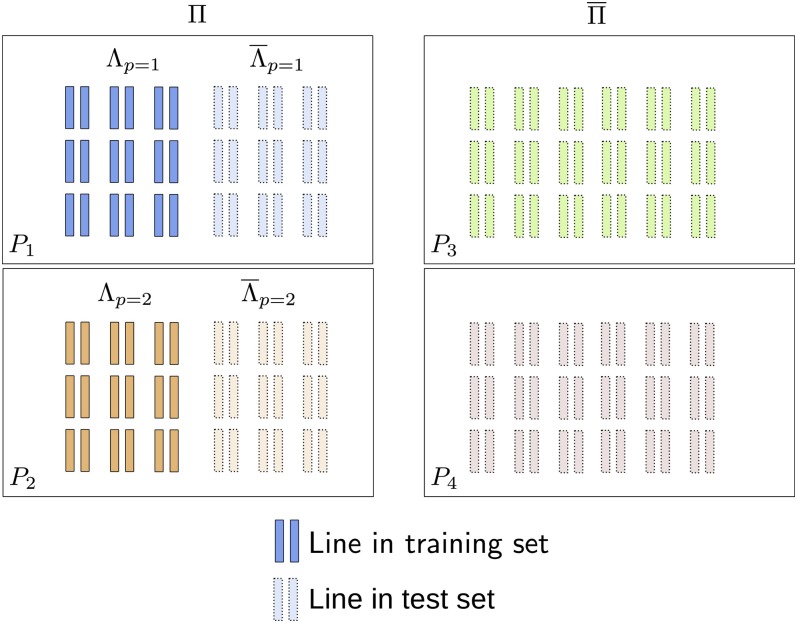
Graphic visualization of the testing strategy for evaluating prediction accuracy. The training set comprises Λ_1_ and Λ_2_ from populations *P*_1_ and *P_2_* (set Π). The prediction accuracy of lines from populations represented in training set ($r_{\Pi }$) was computed from ${\bar{\Lambda }}_{1}$ and ${\bar{\Lambda }}_{2}$, the prediction accuracy of lines from populations not represented in training set from lines in *P*_3_ and *P*_4_ (set $\bar{\Pi }$).

Let Π denote the set of *P* populations represented in the training set and the set of *N_p_* training individuals from a population in Π as *Λ_p_*, where *p* indexes the population in Π. A graphic representation is presented in Figure 2. Further, let those individuals from a population in Π that are not in *Λ_p_* be denoted as ${\bar{\Lambda }}_{p}$ and the set of populations not in Π as $\bar{\Pi }$. Populations in $\bar{\Pi }$ will be referred to as “new” populations. The training set thus comprised all individuals belonging to *Λ_p_*, for p∈Π. The test set used for calculating prediction accuracy, comprised individuals in ${\bar{\Lambda }}_{p}$ from populations in Π and all individuals from populations in $\bar{\Pi }$. The phenotypic observations of test individuals were masked in the estimation procedure. The separation of populations into Π and $\bar{\Pi }$ and of individuals within a population into *Λ_p_* and ${\bar{\Lambda }}_{p}$ was done at random.

Within each population, prediction accuracy was computed as the correlation between GEBVs and observed phenotypic values of individuals in the testing set. The within population prediction accuracies were subsequently averaged for populations in Π and $\bar{\Pi }$. These average within population prediction accuracies will henceforth be denoted as *r*_Π_ and $r_{\bar{\Pi }}$. Thus, *r*_Π_ and $r_{\bar{\Pi }}$ correspond to the prediction accuracy for populations represented and not represented in the training set, respectively.

When using partial pooling, GEBVs of individuals in ${\bar{\Lambda }}_{p}$ were predicted using the posterior means of the marker effects estimated for the corresponding population (*i.e.*, $u_{jk}$). GEBVs of individuals from populations in $\bar{\Pi }$ were predicted using the posterior means of the overall (unspecific) marker effects $u_{k}$.

When using complete pooling, GEBVs of all individuals in the test set were predicted from the posterior means of marker effects $u_{k}$ estimated from the joint data set with model (4).

Finally, when using no pooling, GEBVs of individuals in ${\bar{\Lambda }}_{p}$ were predicted using the posterior means of the marker effects $u_{k}$ obtained after applying model (4) to the training data from the corresponding set *Λ_p_*. The no pooling approach does not provide a direct way of predicting GEBVs of individuals from populations in $\bar{\Pi }$. Thus, $r_{\bar{\Pi }}$ was not evaluated for the no-pooling approach.

### Application to nested association mapping (NAM) maize populations

The NAM data set was obtained from http://www.panzea.org. It comprised 4699 recombinant inbred lines from 25 biparental crosses between a genetically diverse set of maize inbred lines and line B73 as common parent (McMullen *et al.* 2009). The average population size was 188 (range 126–196). The recombinant inbred lines were genotyped with 1106 polymorphic SNP markers covering the whole genome. The non-B73 allele was defined as the reference allele. We confirmed that all SNP were biallelic and thereby that the reference allele corresponded to the same nucleotide in all 25 populations. To facilitate computations, we used a thinned set of 285 markers, chosen in such a way that there was one marker per 5-cM interval, on average. A previous study showed that a density of one marker per 10-cM interval is sufficient for genomic prediction in the NAM population (Guo *et al.* 2012). We analyzed the traits days to silking (DS), ear height (EH), ear length (EL), southern leaf blight resistance (SLB), near-infrared starch measurements (NS) and upper leaf angle (ULA), which were phenotyped in multienvironment field trials. The phenotypic records used for fitting the models were averages over the single environment phenotypes. The number of environments were 10, 11, 8, 3, 7, and 9 for DS, EH, EL, SLB, NS, and ULA, respectively. The traits chosen represent the major trait categories available: yield component (EL), agronomic (EH), disease resistance (SLB), flowering (DS), quality (NS), and morphology (ULA).

To investigate the effect of total number of lines *N*, number of populations *P*, and number of lines per population *N_p_* in the training set on prediction accuracy and the relative performance of the pooling approaches, the following combinations of *P* and *N_p_* were considered: *P* = 5 and *N_p_* = 50 and 100, *P* = 10 and *N_p_* = 25, 50, and 100, *P* = 20 and *N_p_* = 12.5, 25, and 50. For *P* = 20 and *N_p_* = 12.5, we sampled 19 populations with 12 individuals and one with 22, which results in an average *N_p_* of 12.5. The *P* and *N_p_* combinations thus gave rise to *N* of either 250, 500, or 1000. For each combination of trait, *P* and *N_p_*, 50 estimation-testing data sets were generated by repeating the sampling of Π and $\Lambda_{p}$ as described previously. Throughout, the three pooling approaches were applied to the same data sets. The sampling variation between different data sets thus does not enter the comparisons among pooling approaches.

To provide measures of the consistency of differences between pooling approaches under repeated sampling, we plotted the prediction accuracies observed for one pooling approach against those observed for the other in the same 50 data sets. In addition, we conducted paired *t*-tests to assess statistical significance of pairwise differences between pooling approaches.

We conducted an analysis of variance (ANOVA) to assess the overall significance of influencing factors and of interactions between factors on prediction accuracies. For this we fitted a linear model with the main effects of pooling approach, trait, *P* and *N_p_* and all possible interactions among them (*P* and *N_p_* were fitted as numerical predictor variables) and the replication as blocking factor.

To investigate the response to increased marker density, we repeated the analyses using 575 markers, which corresponds to a marker density of 2.5 cM^−1^. Because of the considerably increased central processing unit (CPU) time, particularly for partial pooling (Supporting Information, Figure S10), this was only done for a subset of the traits (EH, EL, and SLB). To facilitate computations for partial pooling, it was also necessary to reduce the length of the thinning interval from 500 to 50 and the number of stored samples from 1000 to 500. The thinning interval length and the number of stored samples for no and complete pooling were not changed, however.

For the purpose of gauging the CPU time requirements with increasing number of markers, we ran the multilevel Gibbs sampler for partial pooling as well as the no and complete pooling BayesA algorithm with numbers of markers from 100 to 1000 in steps of 100. This was done for trait SLB with *P* = 20 and *N_p_* = 25. Because we were only interested in measuring computation time, the Gibbs samplers were run for only 1000 iterations. The whole process was repeated 50 times for each number of markers.

### Application to interconnected biparental (IB) maize populations

This data set was obtained from the supplement of Riedelsheimer *et al.* (2013). It comprised 635 doubled haploid (DH) lines from five biparental populations with average size of 127 (range 43–204). The populations were derived from crosses between four European flint inbred lines. For all DH lines, 16,741 SNP markers polymorphic across populations were available. We replaced missing marker genotypes with twice the frequency of the reference allele, which was the allele with the lower frequency. When analyzing the data we used a thinned set of 285 markers. Because the data set did not include a map of the markers, the markers were chosen randomly.

The DH lines were phenotyped in multienvironment field trials for Giberella ear rot (GER) severity, a fungal disease caused by *Fusarium graminearum*, deoxynivalenol (DON) content (major mycotoxin produced by the fungus), ear length (EL), kernel rows, and kernels per row (KpR). A more detailed description of this data set can be found in Riedelsheimer *et al.* (2013) and Martin *et al.* (2012).

As described previously, populations were randomly split into *Λ_p_* and ${\bar{\Lambda }}_{p}$. However, because there were only five populations in total, we did not exclude any populations from Π. Set $\bar{\Pi }$ was thus empty and we did not evaluate $r_{\bar{\Pi }}$.

The sets *Λ_p_* comprised 25%, 50%, and 75% of the lines in each population, which corresponded to an average *N_p_* of 31, 63, and 95, respectively. For each trait and percentage value of estimation individuals, 100 estimation-testing data sets generated, each time resampling the subset of 285 markers, too.

### Application to simulated data set

We conducted a simulation study to specifically investigate the performance of the pooling approaches under increasing levels of differences in QTL effects among populations. The basis for the simulation were the marker genotypes of the lines in the NAM populations. To simulate genetic values, we first randomly chose 20 marker loci as QTL, which were subsequently removed from the set of observed markers. We drew additive overall effects $a_{q}$ from a standard normal distribution. Then population specific QTL effects $a_{jq}$ were sampled from $N\left(a_{q},\tau_{q}^{2}\right)$. The variance parameter *t*^2^ was chosen such that the relative SD (rSD), *i.e.*, $\tau_{q}/a_{q}$, was equal to 2, 1, 0.5, 0.25, and 0.0. The greater rSD, the less similar the population specific QTL effects are. True genetic values were obtained by summing QTL effects $a_{jq}$ according the QTL genotypes of each individual. Finally, phenotypic values were simulated by adding a normally distributed noise variable to the true genetic values. The variance of the noise variable was chosen such that the heritability across populations was equal to 0.70. The average within family heritability necessarily increased with decreasing rSD, and was 0.53, 0.58, 0.64, 0.68, and 0.70 at rSD 2, 1, 0.5, 0.25, and 0.0, respectively.

Set Π comprised *P* = 10 populations and sets *Λ_p_* had size *N_p_* = 25. For each rSD value 50 training-testing data sets were generated. The QTL positions and effects were randomly generated anew for each data set. Also in this case we used a thinned set of 285 markers. Because the true genetic values were known, $r_{\Pi }$ and $r_{\bar{\Pi }}$ were computed as the correlation between true genetic values and GEBVs.

## Results

### NAM maize populations

Trends typically held across traits. The results presented and discussed therefore apply to all traits, unless otherwise mentioned. We will also present results for 285 markers first and then contrast these with those obtained with 575 markers.

**Figure 3:**
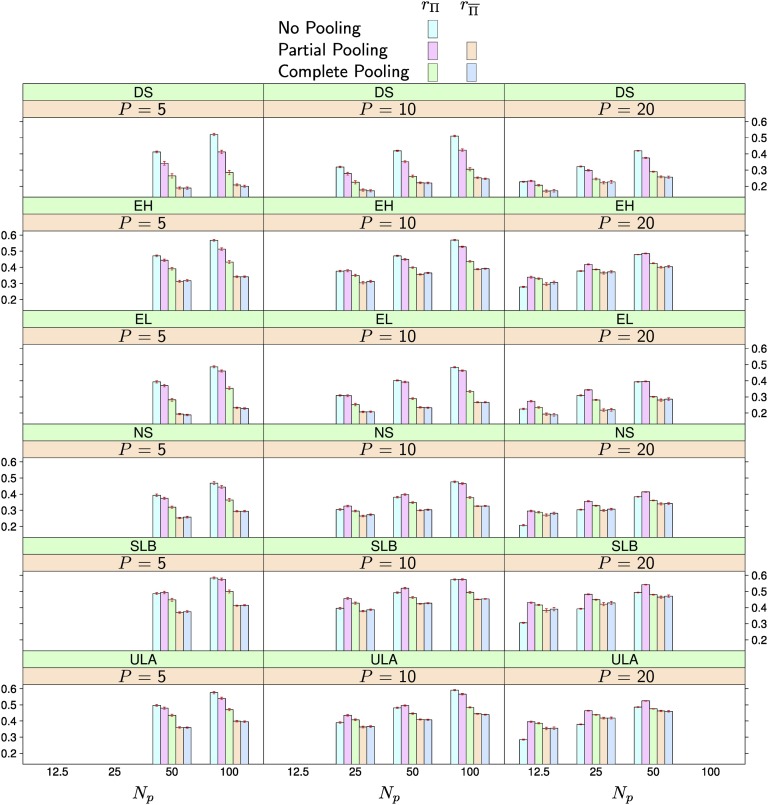
Average prediction accuracy in the nested association mapping population using 285 markers. The number of populations represented in the training set is *P* and the number of individuals per population is *N_p_*. The three leftmost columns in each subplot show the accuracy for no, partial, and complete pooling for populations represented in the training set ($r_{\Pi }$). The two rightmost columns in each subplot show the accuracy for partial and complete pooling for populations not represented in the training set ($r_{\bar{\Pi }}$). The red bars over each column indicate the standard errors of the averages. The traits are: days to silking (DS), ear height (EH), ear length (EL), NIR starch measurements (NS), southern leaf blight resistance (SLB), and upper leaf angle (ULA).

Increasing *N_p_*, while keeping *N* constant (*i.e.*, having fewer but larger populations in the training set) generally increased $r_{\Pi }$ and decreased $r_{\bar{\Pi }}$ (Figure 3 and Table S1). However, the increase in $r_{\Pi }$ was much more pronounced than the decrease in $r_{\bar{\Pi }}$.

When increasing *N_p_* with constant *P* or when increasing *P* with constant *N_p_* (*i.e.*, increasing *N*), both $r_{\Pi }$ and $r_{\bar{\Pi }}$ increased. However, while in the first case, $r_{\Pi }$ and $r_{\bar{\Pi }}$ increased in similar magnitudes, the increase in $r_{\Pi }$ was much smaller than the increase in $r_{\bar{\Pi }}$ in the second case, in particular when *N_p_* was high. Per definition, the accuracy of no pooling is not expected to change as long as *N_p_* remains constant.

For low *P* and high *N_p_*, *e.g.*, *P* = 5 and *N_p_* = 100, no pooling achieved the greatest $r_{\Pi }$ and complete pooling the lowest. For high *P* and low *N_p_*, *e.g.*, P=20 and *N_p_* = 25, partial pooling achieved the greatest $r_{\Pi }$. Here no pooling resulted in the lowest $r_{\Pi }$. The only exception to this was trait DS, where no pooling had a $r_{\Pi }$ equal or higher to partial and complete pooling also for low $N_{p}$. Practically relevant differences among the pooling approaches (*e.g.*, >0.01) were statistically significant (*p* < 0.05, Table S1) and consistent in repeated sampling (Figure S1, Figure S2, Figure S3, Figure S4, Figure S5, Figure S6).

Partial and complete pooling achieved practically identical prediction accuracies $r_{\bar{\Pi }}$ for new populations. In general, $r_{\bar{\Pi }}$ of a particular pooling approach was considerably lower than the corresponding $r_{\Pi }$. The differences between $r_{\Pi }$ and $r_{\bar{\Pi }}$ tended to be larger for high *N_p_*.

In the ANOVA, all main effects and interactions were found to have effects on prediction accuracy that were significantly different than zero (*p* < 0.05), which was expected given the large amount of residual degrees of freedom (Table S2).

**Figure 4:**
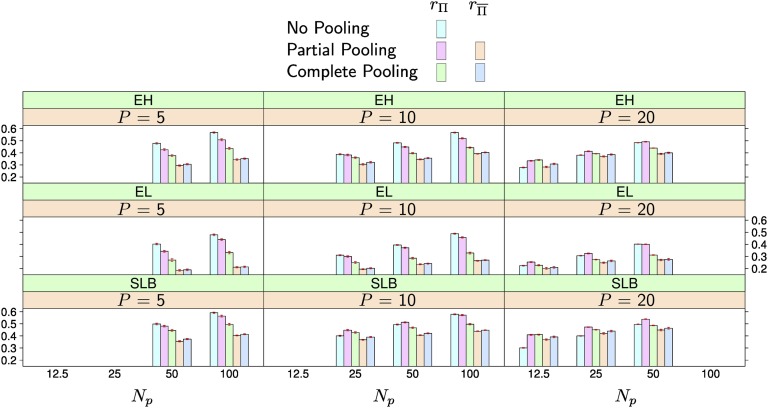
Average prediction accuracy in the nested association mapping population using 575 markers. The number of populations represented in the training set is *P* and the number of individuals per population is *N_p_*. The three leftmost columns in each subplot show the accuracy for no, partial, and complete pooling for populations represented in the training set ($r_{\Pi }$). The two rightmost columns in each subplot show the accuracy for partial and complete pooling for populations not represented in the training set ($r_{\bar{\Pi }}$). The red bars over each column indicate the standard errors of the averages. The traits are: ear height (EH), ear length (EL) and southern leaf blight resistance (SLB).

Results when using 575 markers were similar to those obtained with 285 markers (Figure 4, Figure S7, Figure S8, Figure S9, Table S3 and Table S4) and we did not observe a consistent increase in prediction accuracy. Averaged over traits and *P* and *N_p_* combinations, the difference between 285 and 575 markers were statistically not significant (*p* > 0.05) for $r_{\Pi }$ of no and complete pooling and for $r_{\bar{\Pi }}$ of partial and complete pooling. The only significant difference was observed for $r_{\Pi }$ of partial pooling, which dropped by 0.01 points when increasing the number of markers to 575. It is possible that increased Monte-Carlo error because of the reduced chain length complicated these comparisons, particularly for partial pooling.

CPU time increased linearly with the number of markers for no and complete pooling, but exponentially for partial pooling (Figure S10). Thus, the relative differences between partial pooling on the one hand and no and complete pooling on the other hand increased with increasing number of markers. At 100 markers, the average CPU times for generating 1000 samples were 54, 23, and 2 sec for no, partial, and complete pooling. At 300 markers the corresponding CPU times were 144, 159, and 7 sec and at 1000 markers 503, 1627, and 23 sec.

### IB maize populations

**Figure 5:**
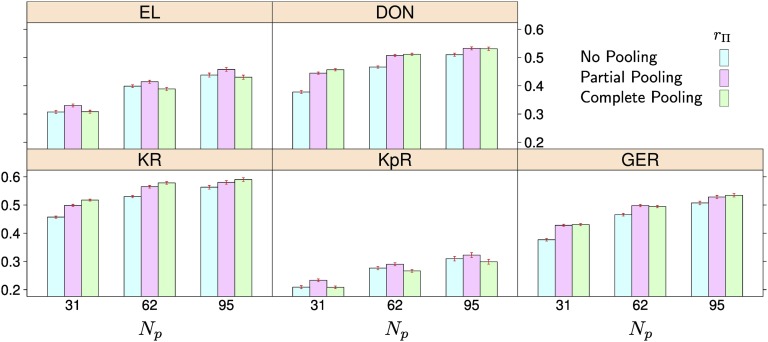
Average prediction accuracies in interconnected biparental maize populations for populations represented in the training set ($r_{\Pi }$). *N_p_* denotes the average number of individuals per population in the training set, the number of populations was 5. The red bars over each column indicate the standard errors of the averages. The traits are: ear length (EL), deoxynivalenol content (DON), Giberella ear rot severity (GER), kernel rows (KR), and kernels per row (KpR).

The prediction accuracy $r_{\Pi }$ increased with increasing *N_p_*, for all traits and pooling approaches (Figure 5 and Table S5). Averaged over traits, the increase was largest for no pooling, where the accuracy increased from an average of 0.35 at *N_p_* = 31 to 0.48 at *N_p_* = 95. The accuracies for the partial and complete pooling approaches increased from 0.39 and 0.38, respectively, at *N_p_* = 31 to 0.48 at *N_p_* = 95.

At *N_p_* = 31, partial pooling had the greatest $r_{\Pi }$for traits EL, KpR, complete pooling for traits DON and kernel row. For GER both had the same accuracy. The no pooling approach had the lowest $r_{\Pi }$, except for EL and KpR, where it had the same accuracy as complete pooling. For the greatest *N_p_* of 95, the accuracy differences among the pooling approaches decreased. Partial pooling still had the greatest accuracy for EL and KpR and the same as complete pooling for DON and GER. While never better than partial pooling, no pooling had higher prediction accuracy than complete pooling for EL and KpR.

Prediction accuracy differences between the pooling approaches were not only statistically significant (*p* < 0.05, Table S5) but also practically relevant (*e.g.*, >0.01) and consistent in repeated sampling (Figure S11, Figure S12, Figure S13, Figure S14, Figure S15). All main effects and interactions were found to have effects on prediction accuracy that were significantly different from zero (*p* < 0.05) in the ANOVA (Table S6).

### Simulated maize populations

**Figure 6:**
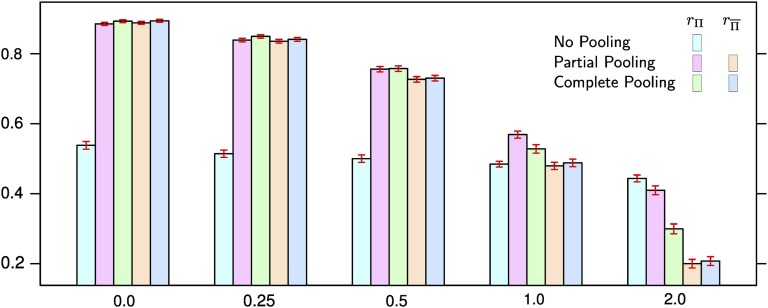
Average prediction accuracy in simulated maize populations. The three leftmost columns in each subplot show the accuracy for no, partial and complete pooling for populations represented in the training set ($r_{\Pi }$). The two rightmost columns in each subplot show the accuracy for partial and complete pooling for populations not represented in the training set ($r_{\bar{\Pi }}$). The red bars over each column indicate the standard errors of the averages. rSD is the relative SD of simulated population specific quantitative trait locus (QTL) effects. The number of populations represented in the training set was 10 and the number of individuals per population 25. The number of markers used was 285 and the number of simulated QTL 20.

For all pooling approaches, $r_{\Pi }$ increased with decreasing rSD (Figure 6 and Table S7). The increase for no pooling, however, was comparatively small and a result of the increasing within family heritability with decreasing rSD. The relative performance of the pooling approaches also depended on rSD. For the greatest rSD value considered, no pooling had the highest $r_{\Pi }$, for the intermediate rSD value of 1.0 partial pooling (Figure S16). For the lower rSD values complete and partial pooling achieved similarly high $r_{\Pi }$.

Also $r_{\bar{\Pi }}$ for both partial and complete pooling increased strongly with decreasing rSD and the differences to $r_{\Pi }$ decreased. Partial and complete pooling achieved almost identical $r_{\bar{\Pi }}$.

All main and interaction effects of factors on prediction accuracy were found to be significantly different from zero (*p* < 0.001) in the ANOVA (Table S8) and prediction accuracy differences between methods were statistically significant (*p* < 0.05, Table S7), practically relevant and consistent in repeated sampling (Figure S16).

The mean of the truncated Normal distribution prior $N{\left(m,d^{2}\right)}^{+}$ for parameter $\gamma_{k}$ increased with increasing rSD. Its average values were 0.0111, 0.0153, 0.0190, 0.0269, and 0.0296 for rSD of 0.0, 0.25, 0.5, 1.0 and 2.0, respectively. The regression coefficient of the regression of the mean of $N{\left(m,d^{2}\right)}^{+}$ on rSD was significantly different from zero (*P* < 0.001).

## Discussion

### Comparison of pooling approaches

Partial pooling allows estimation of population specific marker effects while still facilitating the “borrowing” of information across populations. It is therefore a compromise between no pooling, which models unique characteristics of each population but ignores shared information, and complete pooling, in which the opposite is the case.

When population sizes *N_p_* are sufficiently large, borrowing information from other populations is not required for achieving high prediction accuracy of new individuals from the same population ($r_{\Pi }$). Further enlarging training sets by pooling with other populations might then even be detrimental (Habier *et al.* 2013; Riedelsheimer *et al.* 2013). This explains why no pooling was the most accurate approach when *N_p_* was large (*e.g.*, ≥50), particularly in the NAM population, and why it profited most from increases in *N_p_*. Therefore, pooling of training sets is most promising if *N_p_* is small due to budget or other constraints. We indeed observed that pooling was more accurate than no pooling when *N_p_* was small (*e.g.*, <50). The superiority of either pooling approach over no pooling also increased with increasing *P*, because information from more populations was available, which is not used in no pooling. Thus, pooling is expected to be most advantageous when *P* is relatively high and *N_p_* low. Whether partial or complete pooling is the better approach will then also depend on the similarity of the pooled populations. The greater the similarity, the relatively better complete pooling is expected to perform, because the ability to estimate population specific marker effects becomes less important. In this situation partial pooling might even be of disadvantage, because it requires estimation of many more effects which might lead to problems associated with nonidentifiability (Gelfand and Sahu 1999). The parents of the IB populations are from the same breeding program (Riedelsheimer *et al.* 2013), whereas the noncommon parents of the NAM populations were chosen to be maximally diverse and comprise temperate, tropical and specialty (sweet and popcorn) maize germplasm (McMullen *et al.* 2009). Accommodating for unique characteristics of the populations is therefore more important in NAM than in IB, which might explain why complete pooling was always inferior to partial pooling in the former but often equal or even superior in the latter and also why no pooling never achieved the greatest prediction accuracy in IB, even for large *N_p_*.

The relative performance of the pooling approaches was very stable across traits in the NAM data set, with the exception of DS. For this trait the no pooling approach was generally superior, even at high *P* and low *N_p_*. Buckler *et al.* (2009) found evidence for an allelic series at the QTL identified for DS in the NAM population. Thus, although the positions of the QTL are conserved across populations, their effects might have differed strongly. Possible reasons are presence of multiple alleles or QTL by genetic background interaction. In this situation, pooling of data are not expected to have an advantage over no pooling. This example also shows that decisions about whether to pool data or not have to be made on a by trait basis and should incorporate prior knowledge about genetic architecture, if available.

The dependence of the relative performance of the pooling approaches on the similarity of populations was also reinforced by the results from our simulation study. There we also observed that the mean of $N{\left(m,d^{2}\right)}^{+}$, the prior distribution of $\gamma_{k}$, which quantifies the deviations of specific marker effects $u_{jk}$ from the overall effect $u_{k}$, increased with increasing simulated differences among population specific QTL effects. This was expected, but demonstrates that the data were informative for the high-level hyperparameters. Averaged over *P* and $N_{p}$, this mean was largest for DS and ULA in NAM (results not shown). This might reflect the noted differences between population specific QTL effects for DS. Trait ULA, however, did not diverge from the pattern observed for the remainder of traits and there does not seem to be any strong indication of an allelic series as in DS (Tian *et al.* 2011). There was also no obvious relation between the mean of $N{\left(m,d^{2}\right)}^{+}$ and performance of the pooling approaches in IB (results not shown).

We observed clear trends for the relative performance of the different pooling approaches with regard to changes in *P* and *N_p_*. However, the results showed that factors such as trait and degree of similarity of genetic effects among populations do have an influence on the relative performance. Therefore, deciding *a priori* which pooling approach will be superior for a particular *P* and *N_p_* combination in a given scenario remains challenging and would require substantial prior knowledge about trait architecture and the germplasm.

Modeling unique characteristics of populations requires that these populations are represented in the training set. Prediction of individuals from new populations in $\bar{\Pi }$ therefore has to rely on the overall, unspecific marker effects $u_{k}$, in both partial and complete pooling. It was thus expected that both achieved very similar prediction accuracies $r_{\bar{\Pi }}$ for new populations.

Our results demonstrate that partial pooling is able to model unique characteristics of populations within the training set without compromising on the ability of prediction of individuals from new populations. Thus, our results are consistent with the conclusion of Gelman (2006a), stating that the greatest potential of partial pooling with multilevel models is in predictive applications.

We exemplified the use of multilevel models for partial pooling in the context of multiple populations, a scenario of high relevance for plant (Lehermeier *et al.* 2014) and animal (Lund *et al.* 2014) breeding. However, the concept is readily applicable in a wide array of scenarios. Examples are pooling data across multiple top-cross testers or environments, as is of particular relevance in plant breeding (Albrecht *et al.* 2014). Extending the models to more than two levels is straightforward, too, for example for pooling multiple populations from multiple heterotic groups or breeding programs.

### Computational considerations

For sake of brevity, we will focus on comparing the computational requirements of partial and complete pooling. The CPU time for no pooling could be substantial, and at low number of markers it was even higher than for partial pooling. However, no pooling lends itself to “embarrassingly” parallel computation, because each population is analyzed independently. While parallel execution of analyses in no pooling would not improve total CPU time, it reduces wall time approximately by a factor of *P*. The wall time would then be comparable to complete pooling.

The CPU time for obtaining a certain number of posterior samples was considerably larger for partial pooling than for complete pooling for all numbers of markers investigated. This is partly a consequence of the much greater complexity and dimensionality of multilevel models, in which *P* + 1 effects (specific effects for all population plus the overall effects) have to be estimated for each marker, together with the corresponding variance components. In contrast to no pooling, partial pooling with multilevel models is also not easily parallelized. Most of the prior distributions of hyperparameters used in our multilevel model were not conjugate. This required computationally more demanding sampling techniques such as Metropolis-Hastings within Gibbs. Thus, the choice of prior distributions might have also contributed to the increased computation time.

The software implementation is responsible for the differences in computation time, too. The BayesA algorithm was implemented in the compiled computer language C, while the multilevel model was implemented in JAGS. JAGS, and similar software like BUGS (Thomas *et al.* 2006), are computing environments dedicated to Gibbs sampling and allow straightforward and flexible implementation of novel and complex models. This makes them an ideal choice for research. JAGS, however, is an interpreted language which means program execution is much slower compared to compiled languages. Because the CPU time increased exponentially with increasing number of markers, partial pooling with multilevel models would currently be computationally very intensive for high numbers of markers (*i.e.*, greater than 500).

The populations we studied were all derived from biparental crosses. Previous studies showed that for these types of populations the gain in prediction accuracy is marginal once the number of markers reaches 200–300 (Guo *et al.* 2012; Combs and Bernardo 2013; Hickey *et al.* 2014; Zhang *et al.* 2015). Consequently, we did not observe a significant increase in prediction accuracy for any pooling approach when increasing the number of markers from 285 to 575 (*i.e.*, increasing marker density from 5 cM^−1^ to 2.5 cM^−1^) in the NAM populations.

There are three main reasons why estimating population specific marker effects might be beneficial: (i) inconsistent marker-QTL LD among populations; (ii) QTL by background interaction (Blanc *et al.* 2006; Melchinger *et al.* 2008; Wang *et al.* 2014); and (iii) the presence of genuinely different QTL alleles (Buckler *et al.* 2009; Giraud *et al.* 2014). Of these, only the first is sensitive to increasing marker density. Capturing the genetic variability induced by the other two phenomena requires the use of genetic models that allow marker effects to vary across populations. This can explain why no pooling (when *N_p_* is large) or partial pooling (when *N_p_* is small) had an advantage over complete pooling also for biparental populations, in which marker density might not be the limiting factor.

Biparental crosses are by far the most common source of recombinant germplasm in commercial plant breeding programs. A study by Mikel and Dudley (2006), for example, revealed, that 88% of commercial maize inbred lines developed for the US corn belt between 1980 and 2004 were derived from either F_2_ populations (77%) or backcrosses (11%). The parents of these crosses were in most cases elite and often related inbred lines, with some of them being parent in a disproportionately large number of crosses (Mikel and Dudley 2006). The most prominent example of these is B73, the common parent in the NAM populations. Such key ancestor lines were also identified for the European maize germplasm (Technow *et al.* 2014a). Thus, genomic prediction in sets of biparental populations is of great relevance to plant breeding, and for these types of populations, partial pooling with multilevel models is computationally feasible and can increase prediction accuracy, as we showed.

Nonetheless, there are scenarios where marker density is expected to be a limiting and more decisive factor, for example for pooling dairy cattle breeds (Hayes *et al.* 2009a; Erbe *et al.* 2012) and other types of random mating populations encountered in animal breeding. Application of partial pooling with multilevel models in these cases is more challenging and would require computationally more efficient model formulations and software implementations.

### Alternative approaches to partial pooling

There are alternatives to multilevel models for partial pooling. Brøndum *et al.* (2012) leveraged information across populations by using results obtained from one population to derive genome position specific priors for the analysis of another. For example, when there were two populations A and B, then A was analyzed first and the obtained result used as prior information when analyzing B. One disadvantage of their approach is that because analyses are done sequentially, information is not shared simultaneously among populations. In the example above, information from A is used for B but not vice versa. To use information from B for A, the analyses had to be repeated in reverse order. It is also not obvious how the approach of Brøndum *et al.* (2012) can be generalized to more than two populations or to prediction of individuals from new populations. Another potential source of concern is that the priors derived from population A are too informative to allow substantial Bayesian learning, especially when population B is small (Gelfand and Sahu 1999; Gianola 2013).

Our multilevel model is similar to models that simultaneously fit main and interaction marker effects (Schulz-Streeck *et al.* 2012; De Los Campos and Sorensen 2014). The main difference to our approach is that both effects are on the same hierarchical level, such that the genetic value of an individual is modeled as the sum of main and interaction effects. Nonetheless, both formulations could be viewed as introducing a marginal prior distribution of marker effects that has zero mean and compound symmetric covariance structure (De Los Campos and Sorensen 2014). In multilevel models, however, the overall marker effect $u_{j}$ acts as the prior mean toward which population specific marker effects $u_{jk}$ are shrunk. The magnitude of the shrinkage thereby depends on $\gamma_{k}^{2}$ (the strength of the prior) and on *N_p_* (the “strength” of the data). For very large *N_p_*, the data are expected to diminish the influence of the prior and thereby the shrinkage toward *u_j_*.

Phenotypic observations from different populations could be treated as different traits and pooled data sets analyzed with multi-trait models (De Los Campos and Sorensen 2014; Lund *et al.* 2014). This facilitates simultaneous sharing of information across populations through covariances. Multi-trait extensions of the GBLUP method (*i.e.*, genomic best linear unbiased prediction), which make a strong assumption of uniform variances and covariances of marker effects across the genome (Lund *et al.* 2014), are straight-forward to implement and their performance was evaluated in a dairy cattle breeding context by Karoui *et al.* (2012) and Olson *et al.* (2012). Multitrait extensions of methods like BayesA or BayesB, which would allow for varying covariance structures across markers, are principally conceivable, too (De Los Campos and Sorensen 2014; Lund *et al.* 2014). However, estimating marker specific, unstructured covariance matrices can be expected to be computationally challenging. This is the case particularly when the number of populations is large, because of the increased dimensions of the matrices. In addition, with multitrait models, prediction of performance of individuals from populations not represented in the training set would not be possible directly.

### Composition of training set

Increasing the number of individuals from a population in the training set (*N_p_*) always increased prediction accuracy for untested individuals from the same population ($r_{\Pi }$), regardless if the training set was further enlarged by individuals from other populations (partial and complete pooling) or not (no pooling).

However, because plant breeding programs have to operate under budget constraints, optimum allocation of resources is of great importance for maximizing the potential of genomic selection (Lorenz 2013; Riedelsheimer and Melchinger 2013). With a fixed budget for phenotying that is proportional to *N*, the number of populations *P* and the number of individuals per population *N_p_* have to be optimized under the constraint that $N=P\cdot N_{p}$. Such an optimization could be accomplished using basic theory about response to selection (Falconer and Mackay 1996) and accounting for the different prediction accuracy for populations represented and not represented in the training set ($r_{\Pi }$ and $r_{\bar{\Pi }}$, respectively), as exemplified by Technow *et al.* (2013). A key point hereby is that $r_{\Pi }$ will increase with increasing *N_p_* but it will apply to fewer populations because of the decrease in *P*. This is exacerbated by the decrease in $r_{\bar{\Pi }}$ that we observed was associated with decreasing *P*. Thus, if the total number of populations is large, as is typically the case in plant breeding programs, having very low *P* is likely to be undesirable. In the context of plant breeding this and other studies, most recently Lehermeier *et al.* (2014), showed that pooling data across populations can at least partly compensate for low *N_p_* if populations are related and there is evidence for the merit of pooling very divergent germplasm too (Technow *et al.* 2013). Using pooled training sets therefore has the potential to allow for high *P* without compromising too much on $r_{\Pi }$. We showed that partial pooling with multilevel models can further enhance this potential by making optimal use of the information in pooled training sets.

## 

## Supplementary Material

Supporting Information
