# Supplementary material for: Using Bayesian Multilevel Whole Genome Regression Models for Partial Pooling of Training Sets in Genomic Prediction
Source: G3 (Bethesda). 2015 May 29;5(8):1603–12. doi: 10.1534/g3.115.019299 (PMC4528317; doi:10.1534/g3.115.019299)
Supplement: Supporting Information [file supp_5_8_1603__index.html]

Using Bayesian Multilevel Whole Genome Regression Models for Partial Pooling of Training Sets in Genomic Prediction — Supporting Information 

# Using Bayesian Multilevel Whole Genome Regression Models for Partial Pooling of Training Sets in Genomic Prediction

## Supporting Information for Technow and Totir, 2015

**Files in this Data Supplement:**

- Supporting Information - Tables S1-S8 and Figures S1-S16 (PDF, 6 MB)
- Table S1 - Average within population prediction accuracies in NAM maize populations with 285 markers. (PDF, 75 KB)
- Table S2 - Anova for the influence of factors on prediction accuracy of populations represented in the training set (*r*Π) for the NAM populations with 285 markers. (PDF, 54 KB)
- Table S3 - Average within population prediction accuracies in NAM maize populations using 575 markers. (PDF, 73 KB)
- Table S4 - Anova for the influence of factors on prediction accuracy of populations represented in the training set (*r*Π) for the NAM populations with 575 markers. (PDF, 54 KB)
- Table S5 - Average within population prediction accuracies in interconnected biparental maize populations. (PDF, 55 KB)
- Table S6 - Anova for the influence of factors on prediction accuracy of populations represented in the training set (*r*Π) for the interconnected biparental maize populations. (PDF, 54 KB)
- Table S7 - Average prediction accuracies for simulated maize populations. (PDF, 63 KB)
- Table S8 - Anova for the influence of factors on prediction accuracy of populations represented in the training set (*r*Π) for the simulated maize populations. (PDF, 47 KB)
- Figure S1 - Prediction accuracy *r*Π (for populations represented in the training set) for trait days to silking in the NAM population. (PDF, 481 KB)
- Figure S2 - Prediction accuracy *r*Π (for populations represented in the training set) for trait ear height in the NAM population. (PDF, 494 KB)
- Figure S3 - Prediction accuracy *r*Π (for populations represented in the training set) for trait ear length in the NAM population. (PDF, 474 KB)
- Figure S4 - Prediction accuracy *r*Π (for populations represented in training set) for trait NIR starch measurements in the NAM population. (PDF, 496 KB)
- Figure S5 - Prediction accuracy *r*Π (for populations represented in training set) for trait southern leaf blight in the NAM population. (PDF, 487 KB)
- Figure S6 - Prediction accuracy *r*Π (for populations represented in training set) for trait upper leaf angle in the NAM population. (PDF, 494 KB)
- Figure S7 - Prediction accuracy *r*Π (for populations represented in training set) for trait ear height in the NAM population. (PDF, 504 KB)
- Figure S8 - Prediction accuracy *r*Π (for populations represented in training set) for trait ear length in the NAM population. (PDF, 472 KB)
- Figure S9 - Prediction accuracy *r*Π (for populations represented in training set) for trait southern leaf blight in the NAM population. (PDF, 483 KB)
- Figure S10 - Average CPU time in seconds per 1000 posterior samples for pooling approaches with increasing number of markers. (PDF, 78 KB)
- Figure S11 - Prediction accuracy *r*Π (for populations represented in the training set) for trait ear length in the interconnected biparental maize population. (PDF, 278 KB)
- Figure S12 - Prediction accuracy *r*Π (for populations represented in the training set) for trait deoxinivalenol content in the interconnected biparental maize population. (PDF, 291 KB)
- Figure S13 - Prediction accuracy *r*Π (for populations represented in the training set) for trait Giberella ear rot severity in the interconnected biparental maize populations. (PDF, 287 KB)
- Figure S14 - Prediction accuracy *r*Π (for populations represented in the training set) for trait kernel rows in the interconnected biparental maize populations. (PDF, 285 KB)
- Figure S15 - Prediction accuracy *r*Π (for populations represented in the training set) for trait kernels per row interconnected biparental maize populations. (PDF, 282 KB)
- Figure S16 - Prediction accuracy *r*Π (for populations represented in the training set) in simulated maize populations. (PDF, 322 KB)
