# Supplementary material for: Using Bayesian Multilevel Whole Genome Regression Models for Partial Pooling of Training Sets in Genomic Prediction
Source: G3 (Bethesda). 2015 May 29;5(8):1603–12. doi: 10.1534/g3.115.019299 (PMC4528317; doi:10.1534/g3.115.019299)
Supplement: Supporting Information [file supp_g3.115.019299_019299SI.pdf]

G3 · Genes | Genomes | Genetics

Using Bayesian multilevel whole genome regression models for partial pooling of training sets in genomic prediction

Frank Technow and L. Radu Totir

DuPont Pioneer, Johnston, Iowa 50131

Corresponding Author:
Frank Technow
DuPont Pioneer
8305 NW 62nd Ave
Johnston, Iowa 50131
Frank.Technow@pioneer.com
Tel.: +1 (515) 535-8317
Fax: +1 (515) 535-0226

DOI: 10.1534/g3.115.019299

TABLE S1: Average within population prediction accuracies in NAM maize populations with 285 markers

			r_{Π}			$r_{\overline{\Pi}}$	
P	N_p	trait	no pooling	partial pooling	complete pooling	partial pooling	complete pooling
5	50	DS	0.41[a]	0.34[b]	0.2[c]	0.19[a]	0.19[a]
		EH	0.47[a]	0.44[b]	0.39[c]	0.31[a]	0.32[b]
		EL	0.39[a]	0.37[b]	0.28[c]	0.19[a]	0.19[b]
		NS	0.39[a]	0.37[b]	0.32[c]	0.25[a]	0.26[b]
		SLB	0.49[a]	0.49[a]	0.45[b]	0.37[a]	0.37[b]
		ULA	0.50[a]	0.48[b]	0.44[c]	0.36[a]	0.36[a]
	100	DS	0.52[a]	0.41[b]	0.28[c]	0.21[a]	0.20[b]
		EH	0.57[a]	0.51[b]	0.43[c]	0.34[a]	0.34[a]
		EL	0.49[a]	0.46[b]	0.35[c]	0.23[a]	0.23[b]
		NS	0.47[a]	0.44[b]	0.36[c]	0.29[a]	0.29[a]
		SLB	0.58[a]	0.58[a]	0.50[b]	0.41[a]	0.41[a]
		ULA	0.58[a]	0.54[b]	0.47[c]	0.40[a]	0.40[a]
10	25	DS	0.32[a]	0.28[b]	0.22[c]	0.18[a]	0.17[b]
		EH	0.38[a]	0.38[a]	0.35[b]	0.30[a]	0.31[b]
		EL	0.31[a]	0.31[a]	0.25[b]	0.21[a]	0.21[a]
		NS	0.30[a]	0.33[b]	0.30[a]	0.26[a]	0.27[b]
		SLB	0.40[a]	0.46[b]	0.43[c]	0.38[a]	0.39[b]
		ULA	0.39[a]	0.44[b]	0.41[c]	0.36[a]	0.37[a]
	50	DS	0.42[a]	0.35[b]	0.26[c]	0.22[a]	0.22[a]
		EH	0.47[a]	0.45[b]	0.40[c]	0.36[a]	0.36[b]
		EL	0.40[a]	0.39[b]	0.29[c]	0.23[a]	0.23[a]
		NS	0.38[a]	0.40[b]	0.35[c]	0.30[a]	0.30[b]
		SLB	0.49[a]	0.52[b]	0.46[c]	0.42[a]	0.43[b]
		ULA	0.48	0.50	0.45	0.41[a]	0.41[a]
	100	DS	0.51[a]	0.42[b]	0.30[c]	0.25[a]	0.25[b]
		EH	0.57[a]	0.53[b]	0.44[c]	0.39[a]	0.39[b]
		EL	0.48[a]	0.46[b]	0.33[c]	0.27[a]	0.27[a]
		NS	0.48[a]	0.46[b]	0.38[c]	0.33[a]	0.33[a]
		SLB	0.57[a]	0.57[a]	0.49[b]	0.45[a]	0.45[b]
		ULA	0.59[a]	0.56[b]	0.48[c]	0.45[a]	0.44[b]
20	12.5	DS	0.23[a]	0.23[a]	0.21[b]	0.17[a]	0.17[a]
		EH	0.28[a]	0.34[b]	0.33[c]	0.30[a]	0.31[b]
		EL	0.22[a]	0.27[b]	0.23[a]	0.19[a]	0.19[a]
		NS	0.21[a]	0.30[b]	0.29[c]	0.27[a]	0.28[a]
		SLB	0.31[a]	0.43[b]	0.42[c]	0.38[a]	0.39[a]
		ULA	0.28[a]	0.40[b]	0.39[c]	0.35[a]	0.36[a]
	25	DS	0.32[a]	0.30[b]	0.24[c]	0.22[a]	0.23[a]
		EH	0.38[a]	0.42[b]	0.39[c]	0.36[a]	0.37[b]

Continued on next page

	EL	0.31^{a}	0.34^{b}	0.28^{c}	0.22^{a}	0.22^{a}
	NS	0.30^{a}	0.36^{b}	0.33^{b}	0.30^{a}	0.31^{b}
	SLB	0.39^{a}	0.48^{b}	0.45^{c}	0.42^{a}	0.43^{b}
	ULA	0.38^{a}	0.46^{b}	0.44^{c}	0.42^{a}	0.42^{a}
50	DS	0.42^{a}	0.37^{b}	0.29^{c}	0.26^{a}	0.26^{a}
	EH	0.48^{a}	0.49^{b}	0.42^{c}	0.40^{a}	0.40^{a}
	EL	0.39^{a}	0.40^{a}	0.30^{b}	0.28^{a}	0.29^{b}
	NS	0.38^{a}	0.41^{b}	0.36^{c}	0.34^{a}	0.34^{a}
	SLB	0.49^{a}	0.54^{b}	0.48^{c}	0.46^{a}	0.47^{b}
	ULA	0.49^{a}	0.52^{b}	0.47^{c}	0.46^{a}	0.46^{a}

Values shown are average within population prediction accuracies for test individuals, averaged over 50 random estimation-test data splits. Values with common letters within a row and within either r_{Π} or $r_{\overline{\Pi}}$ are not significantly different in paired t-tests at an alpha level of 0.05. The standard errors of the averages were $<$ 0.013. r_{Π} is the prediction accuracy for populations represented in the training set and $r_{\overline{\Pi}}$ the prediction accuracy of populations not represented in the training set. P gives the size of set Π, i.e., the number of populations represented in the training set, column N_p gives the number of individuals from each population in Π that were used for estimation, i.e., the sizes of sets Λ_p. The traits are: days to silking (DS), ear height (EH), ear length (EL), southern leaf blight resistance (SLB), near-infrared starch measurements (NS) and upper leaf angle (ULA).

TABLE S2: Anova for the influence of factors on prediction accuracy of populations represented in the training set (r_{Π}) for the NAM populations with 285 markers

Source	Df	Sum Sq	Mean Sq	F value	Pr(>F)
pooling	2	5.23	2.62	3223.38	0.0000
trait	5	24.06	4.81	5931.88	0.0000
N_p	1	22.58	22.58	27824.73	0.0000
P	1	0.07	0.07	86.06	0.0000
replication	2391	10.90	0.00	5.62	0.0000
pooling:trait	10	2.52	0.25	310.73	0.0000
pooling:N_p	2	3.92	1.96	2417.16	0.0000
pooling:P	2	0.38	0.19	233.29	0.0000
pooling:trait:N_p	10	0.08	0.01	10.08	0.0000
pooling:trait:P	10	0.02	0.00	2.15	0.0178
pooling:N_p:P	2	0.23	0.11	141.71	0.0000
pooling:trait:N_p:P	10	0.02	0.00	1.95	0.0345
Residuals	4750	3.85	0.00		

Degrees of freedom (Df), sum of squares (Sum Sq), mean squares (Mean Sq). The pooling approaches are referred to as ‘pooling’

TABLE S3: Average within population prediction accuracies in NAM maize populations using 575 markers

			r_{Π}			$r_{\overline{\Pi}}$	
P	N_p	trait	no pooling	partial pooling	complete pooling	partial pooling	complete pooling
5	50	EH	0.48^{a}	0.43^{b}	0.38^{c}	0.29^{a}	0.31^{b}
		EL	0.40^{a}	0.34^{b}	0.27^{c}	0.19^{a}	0.19^{a}
		SLB	0.50^{a}	0.48^{b}	0.45^{c}	0.36^{a}	0.37^{b}
	100	EH	0.57^{a}	0.51^{b}	0.44^{c}	0.34^{a}	0.35^{b}
		EL	0.48^{a}	0.44^{b}	0.33^{c}	0.21^{a}	0.21^{a}
		SLB	0.59^{a}	0.56^{b}	0.50^{c}	0.40^{a}	0.41^{b}
10	25	EH	0.39^{a}	0.38^{a}	0.36^{b}	0.30^{a}	0.32^{b}
		EL	0.31^{a}	0.30^{a}	0.25^{b}	0.20^{a}	0.20^{b}
		SLB	0.40^{a}	0.45^{b}	0.43^{c}	0.37^{a}	0.39^{b}
	50	EH	0.48^{a}	0.45^{b}	0.40^{c}	0.35^{a}	0.36^{b}
		EL	0.39^{a}	0.37^{b}	0.29^{c}	0.24^{a}	0.24^{b}
		SLB	0.49^{a}	0.51^{b}	0.47^{c}	0.41^{a}	0.42^{b}
	100	EH	0.57^{a}	0.52^{b}	0.44^{c}	0.39^{a}	0.40^{b}
		EL	0.49^{a}	0.46^{b}	0.33^{c}	0.26^{a}	0.27^{b}
		SLB	0.58^{a}	0.57^{b}	0.50^{c}	0.44^{a}	0.45^{b}
20	12.5	EH	0.28^{a}	0.33^{b}	0.34^{c}	0.28^{a}	0.31^{b}
		EL	0.22^{a}	0.25^{b}	0.23^{a}	0.20^{a}	0.21^{b}
		SLB	0.30^{a}	0.41^{b}	0.41^{b}	0.37^{a}	0.39^{b}
	25	EH	0.38^{a}	0.41^{b}	0.39^{c}	0.37^{a}	0.39^{b}
		EL	0.31^{a}	0.32^{b}	0.27^{c}	0.25^{a}	0.26^{b}
		SLB	0.40^{a}	0.47^{b}	0.45^{c}	0.42^{a}	0.44^{b}
	50	EH	0.48^{a}	0.49^{b}	0.44^{c}	0.39^{a}	0.40^{b}
		EL	0.40^{a}	0.40^{a}	0.31^{b}	0.27^{a}	0.28^{a}
		SLB	0.50^{a}	0.54^{b}	0.49^{c}	0.45^{a}	0.46^{b}

Values shown are average within population prediction accuracies for test individuals, averaged over 50 random estimation-test data splits. Values with common letters within a row and within either $r\Pi$ or $r_{\overline{\Pi}}$ are not significantly different in paired t-tests at an alpha level of 0.05. The standard errors of the averages were $<$ 0.01. r_{Π} is the prediction accuracy for populations represented in the training set and $r_{\overline{\Pi}}$ the prediction accuracy of populations not represented in the training set. P gives the size of set Π, i.e., the number of populations represented in the training set, column N_p gives the number of individuals from each population in Π that were used for estimation, i.e., the sizes of sets Λ_p. The traits were: ear height (EH), ear length (EL) and southern leaf blight resistance (SLB).

TABLE S4: Anova for the influence of factors on prediction accuracy of populations represented in the training set (r_{Π}) for the NAM populations with 575 markers

Source	Df	Sum Sq	Mean Sq	F value	Pr(>F)
pooling	2	2.19	1.10	1541.96	0.0000
trait	2	11.49	5.75	8084.31	0.0000
N_p	1	11.38	11.38	16016.89	0.0000
P	1	0.09	0.09	126.11	0.0000
replication	1195	5.46	0.00	6.43	0.0000
pooling:trait	4	0.72	0.18	254.16	0.0000
pooling:N_p	2	1.82	0.91	1283.11	0.0000
pooling:P	2	0.30	0.15	207.80	0.0000
pooling:trait:N_p	4	0.04	0.01	12.82	0.0000
pooling:trait:P	4	0.01	0.00	2.15	0.0718
pooling:N_p:P	2	0.13	0.07	94.45	0.0000
pooling:trait:N_p:P	4	0.00	0.00	0.33	0.8561
Residuals	2376	1.69	0.00		

Degrees of freedom (Df), sum of squares (Sum Sq), mean squares (Mean Sq). The pooling approaches are referred to as “pooling”

TABLE S5: Average within population prediction accuracies in interconnected biparental maize populations

N_p	Trait	Pooling		
		no	partial	complete
31	EL	0.31[a]	0.33[b]	0.31[a]
	DON	0.38[a]	0.44[b]	0.46[c]
	GER	0.38[a]	0.43[b]	0.43[b]
	KR	0.46[a]	0.50[b]	0.52[c]
	KpR	0.21[a]	0.23[b]	0.21[a]
62	EL	0.40[a]	0.40[b]	0.39[a]
	DON	0.47[a]	0.51[b]	0.51[c]
	GER	0.47[a]	0.50[b]	0.49[b]
	KR	0.53[a]	0.56[b]	0.58[c]
	KpR	0.28[a]	0.29[b]	0.27[a]
95	EL	0.44[a]	0.46[b]	0.43[a]
	DON	0.51[a]	0.53[b]	0.53[b]
	GER	0.51[a]	0.53[b]	0.53[c]
	KR	0.56[a]	0.58[b]	0.59[c]
	KpR	0.31[a]	0.32[b]	0.30[a]

Values shown are average within population prediction accuracies for test individuals, averaged over 100 random estimation-test data splits. Values within a row with common letters are not significantly different at an alpha level of 0.05 in a paired t-test. Standard errors of the averages were $<$ 0.01. N_p denotes the average number of individuals per population in the training set. The traits were ear length (EL), deoxynivalenol content (DON), Giberella ear rot severity (GER) kernel rows (KR) and kernels per row (KpR)

TABLE S6: Anova for the influence of factors on prediction accuracy of populations represented in the training set (r_{Π}) for the interconnected biparental maize populations

Source	Df	Sum Sq	Mean Sq	F value	Pr(>F)
pooling	2	0.72	0.36	397.65	0.0000
trait	4	40.67	10.17	11260.24	0.0000
N_p	1	8.01	8.01	8866.52	0.0000
replication	1494	10.99	0.01	8.14	0.0000
pooling:trait	8	0.48	0.06	66.38	0.0000
pooling:N_p	2	0.11	0.05	59.21	0.0000
pooling:trait:N_p	8	0.05	0.01	6.56	0.0000
Residuals	2980	2.69	0.00		

Degrees of freedom (Df), sum of squares (Sum Sq), mean squares (Mean Sq). The pooling approaches are referred to as “pooling”

TABLE S7: Average prediction accuracies for simulated maize populations

	r_{Π}			$r_{\overline{\Pi}}$	
rSD	no pooling	partial pooling	complete pooling	partial pooling	complete pooling
0.0	0.54[a]	0.89[b]	0.89[c]	0.89[a]	0.89[b]
0.25	0.51[a]	0.84[b]	0.85[c]	0.84[a]	0.84[b]
0.5	0.50[a]	0.76[b]	0.76[b]	0.73[a]	0.73[b]
1.0	0.48[a]	0.57[b]	0.53[c]	0.48[a]	0.49[b]
2.0	0.44[a]	0.41[b]	0.30[c]	0.20[a]	0.21[b]

Values shown are average within population prediction accuracies for test individuals, averaged over 50 random estimation-test data splits. Standard errors were < 0.015. r_{Π} is the prediction accuracy for populations represented in the training set and $r_{\overline{\Pi}}$ the prediction accuracy of populations not represented in the training set. rSD is the relative standard deviation of simulated population specific QTL effects.

TABLE S8: Anova for the influence of factors on prediction accuracy of populations represented in the training set (r_{Π}) for the simulated maize populations

Source	Df	Sum Sq	Mean Sq	F value	Pr(>F)
pooling	2	7.14	3.57	1786.49	0.0000
rSD	5	17.04	3.41	1706.58	0.0000
replication	269	2.36	0.01	4.39	0.0000
pooling:rSD	10	5.41	0.54	270.67	0.0000
Residuals	538	1.07	0.00		

Degrees of freedom (Df), sum of squares (Sum Sq), mean squares (Mean Sq). The pooling approaches are referred to as "pooling", rSD is the relative standard deviation of simulated population specific QTL effects

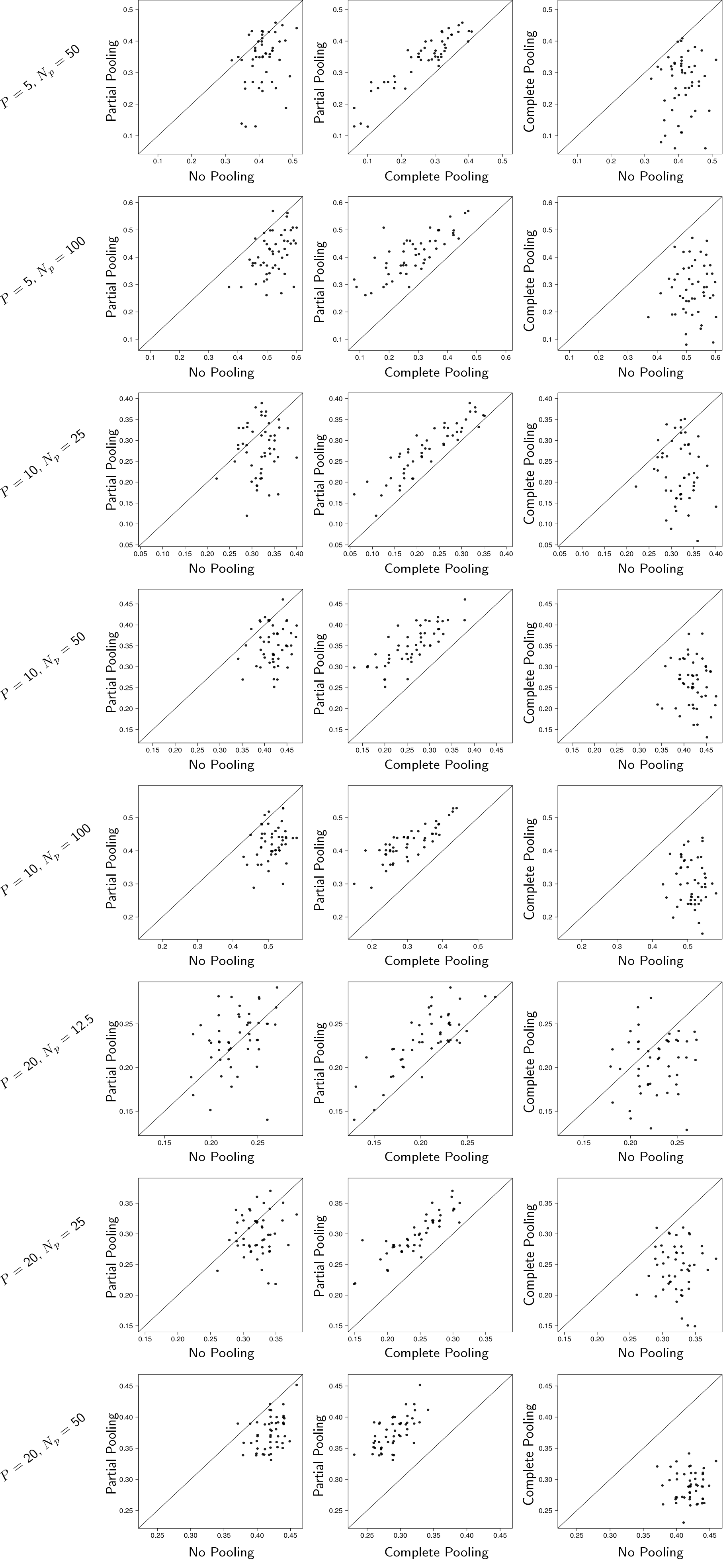

Figure S1 Prediction accuracy r_{Π} (for populations represented in training set) for trait days to silking in the NAM population. The points correspond to the replications of the cross-validation. The number of populations in the training set is P and the number of individuals per population is N_p. The number of markers used was 285.

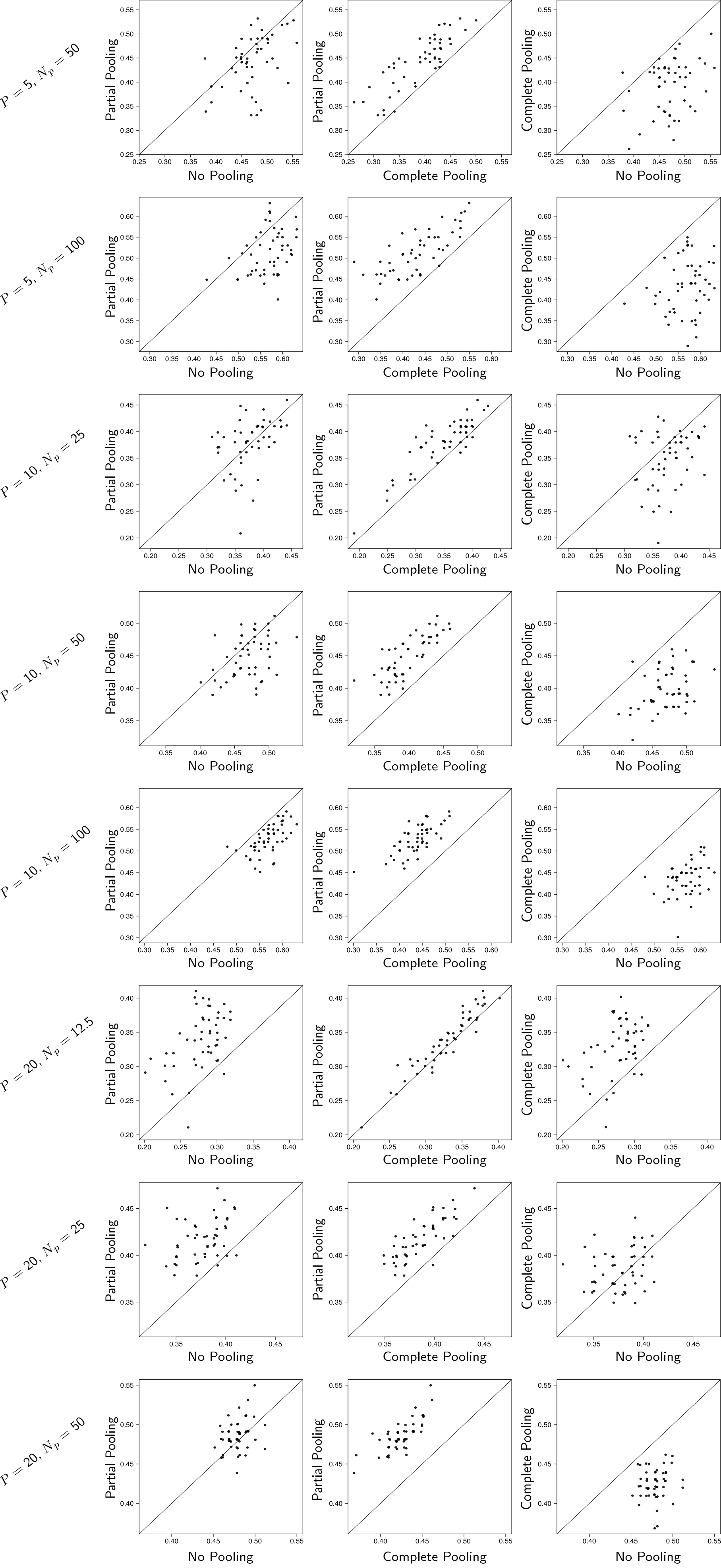

Figure S2 Prediction accuracy r_{Π} (for populations represented in training set) for trait ear height in the NAM population. The points correspond to the replications of the cross-validation. The number of populations in the training set is P and the number of individuals per population is N_p. The number of markers used was 285.

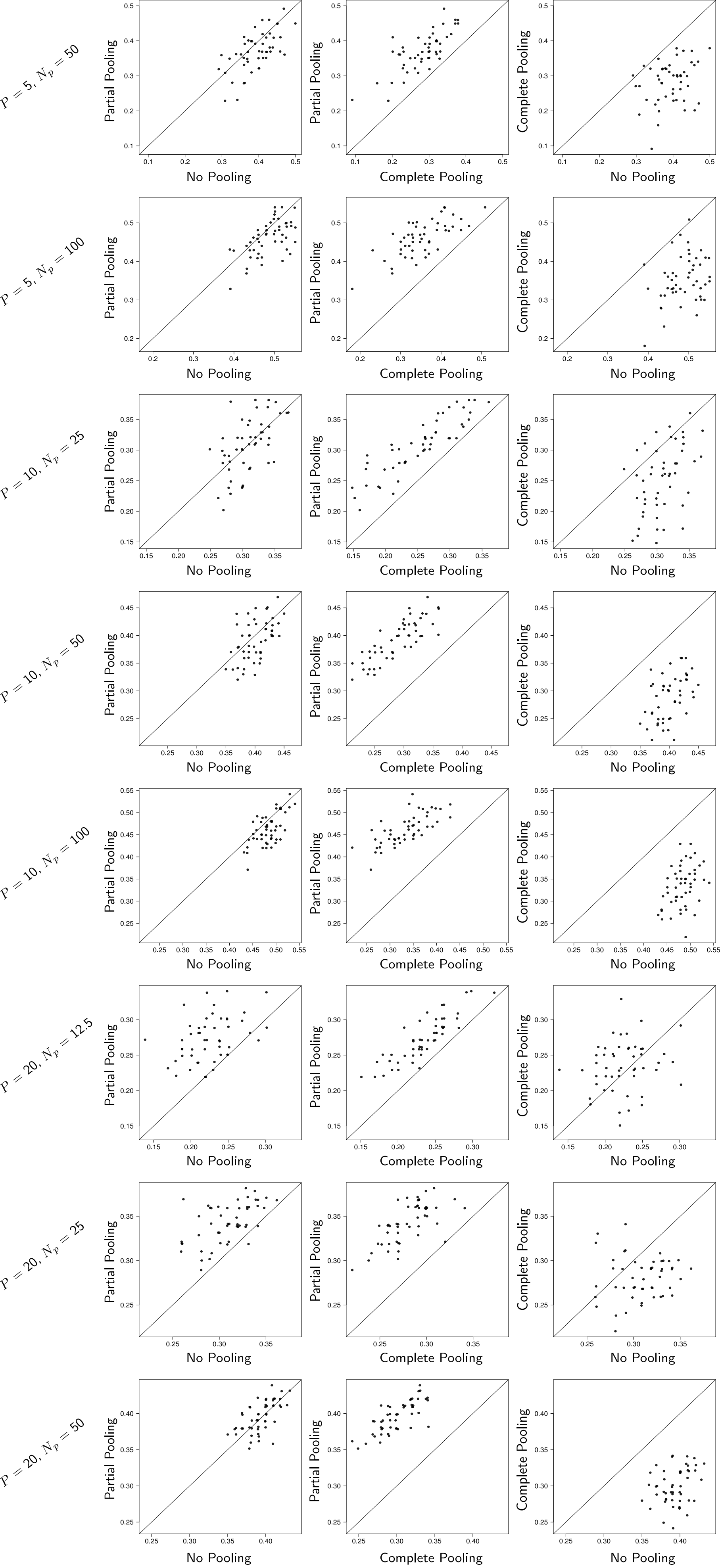

Figure S3 Prediction accuracy r_{Π} (for populations represented in training set) for trait ear length in the NAM population. The points correspond to the replications of the cross-validation. The number of populations in the training set is P and the number of individuals per population is N_p. The number of markers used was 285.

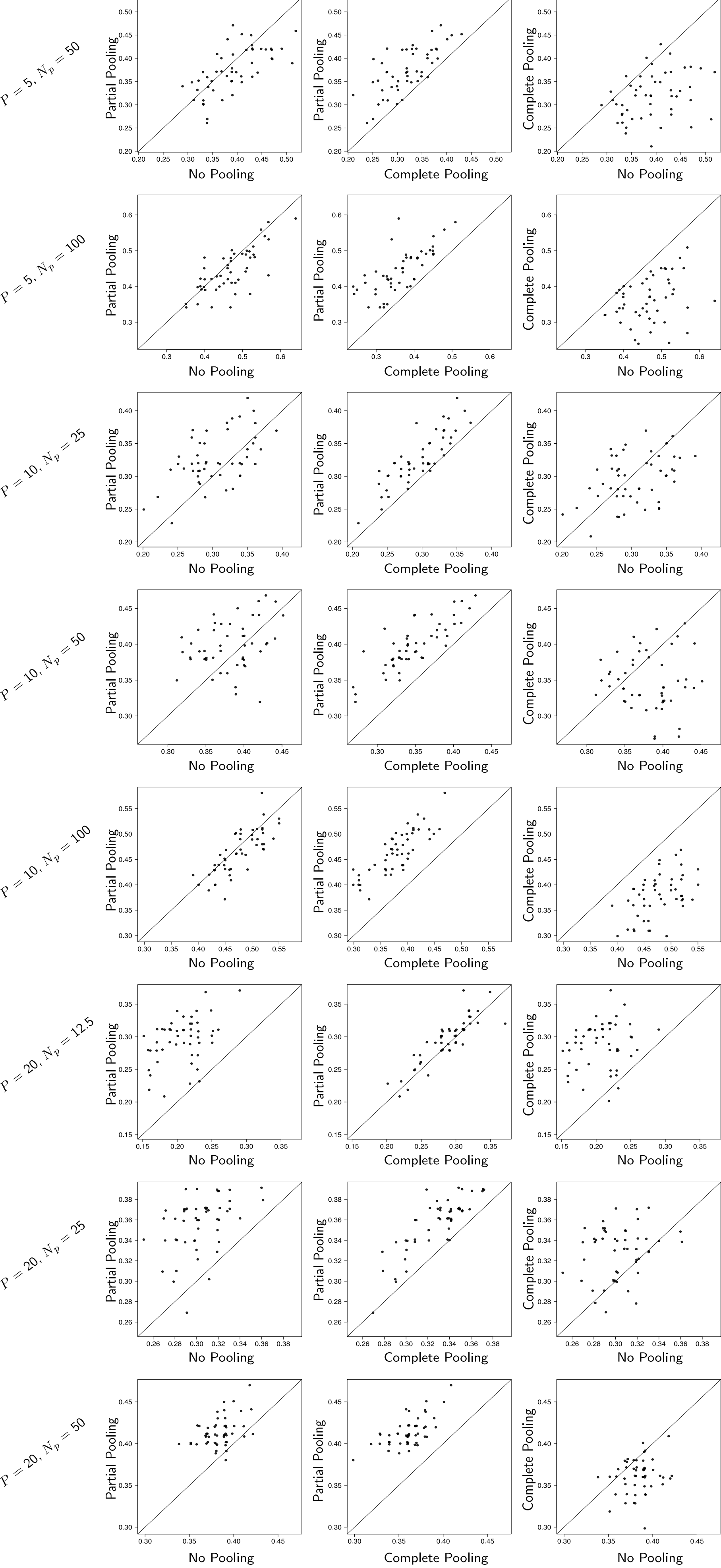

Figure S4 Prediction accuracy r_{Π} (for populations represented in training set) for trait NIR starch measurements in the NAM population. The points correspond to the replications of the cross-validation. The number of populations in the training set is P and the number of individuals per population is N_p. The number of markers used was 285.

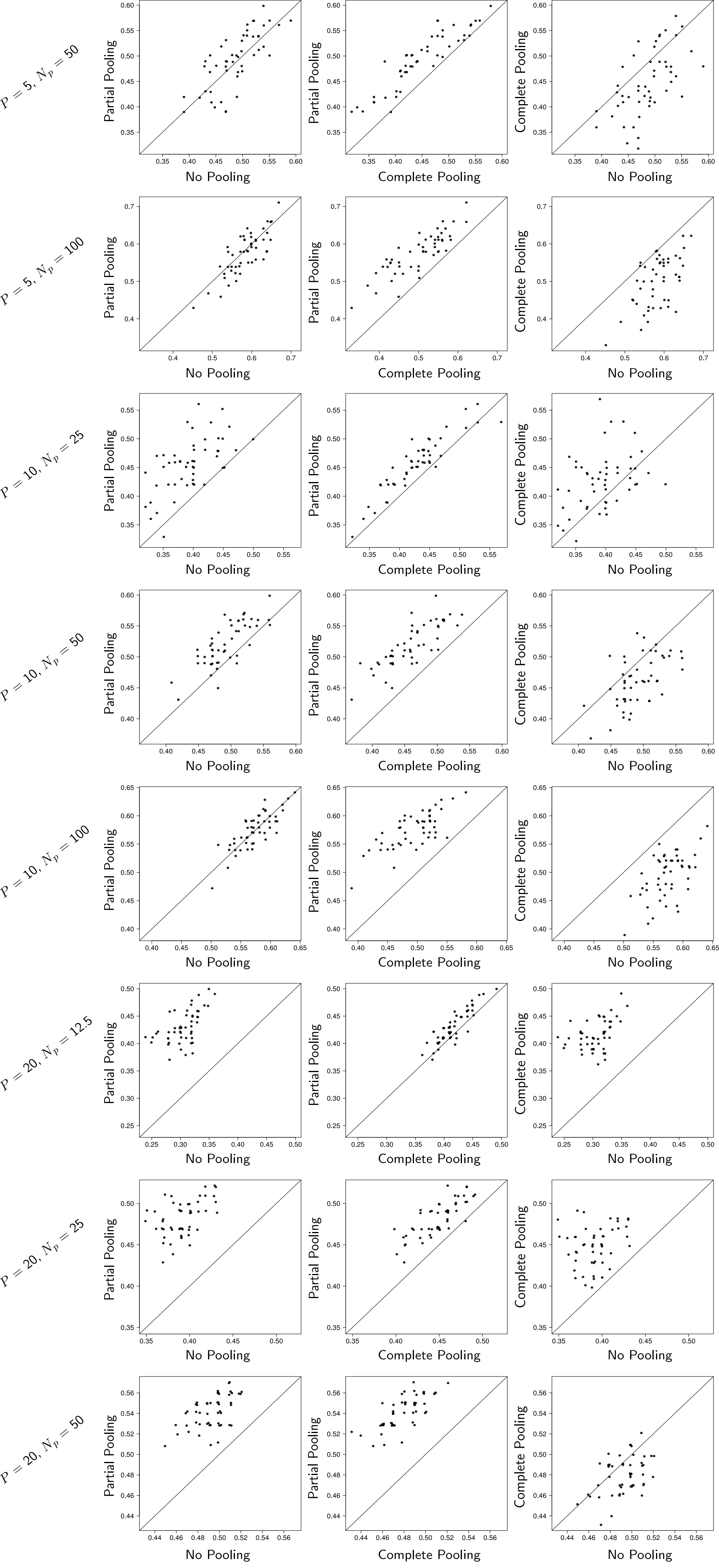

Figure S5 Prediction accuracy r_{Π} (for populations represented in training set) for trait southern leaf blight in the NAM population. The points correspond to the replications of the cross-validation. The number of populations in the training set is P and the number of individuals per population is N_p. The number of markers used was 285.

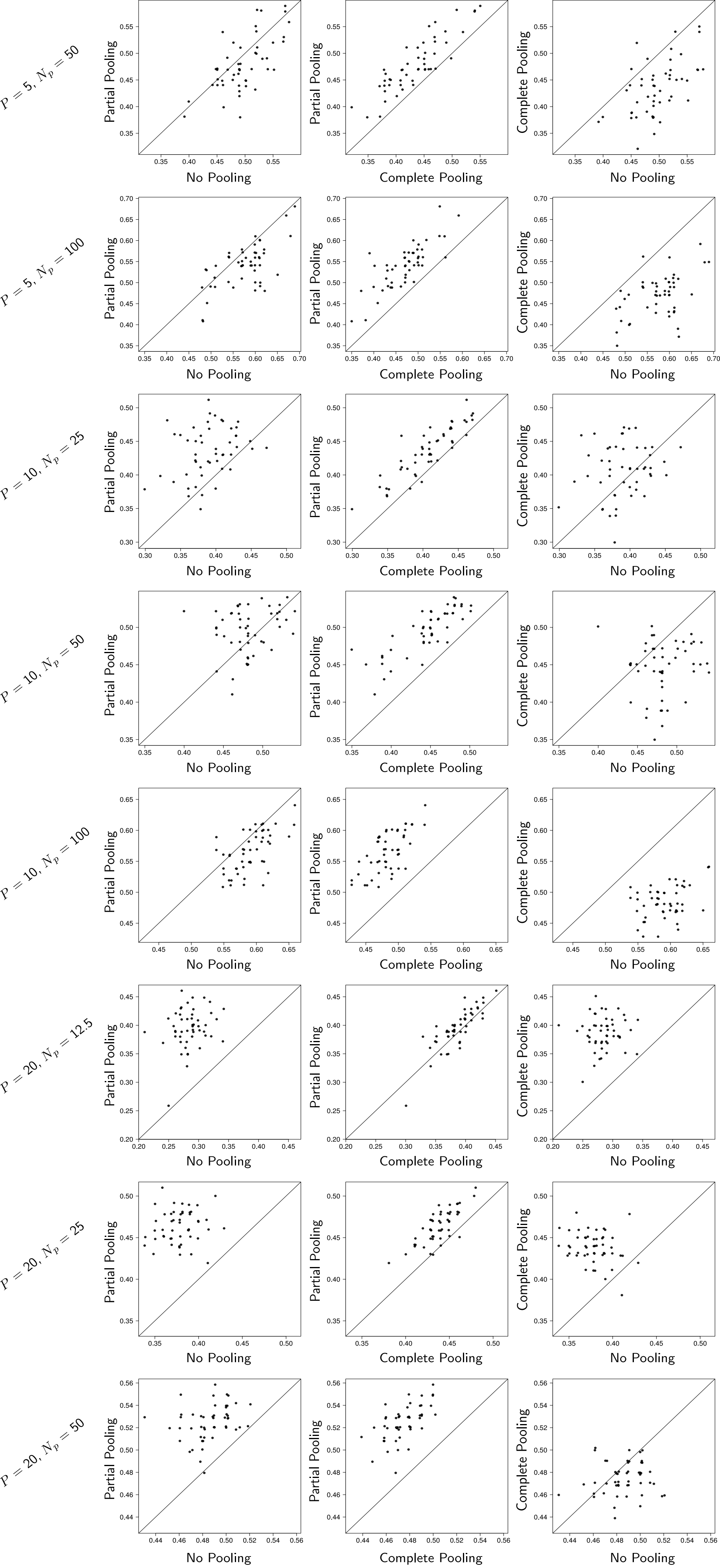

Figure S6 Prediction accuracy r_{Π} (for populations represented in training set) for trait upper leaf angle in the NAM population. The points correspond to the replications of the cross-validation. The number of populations in the training set is P and the number of individuals per population is N_p. The number of markers used was 285.

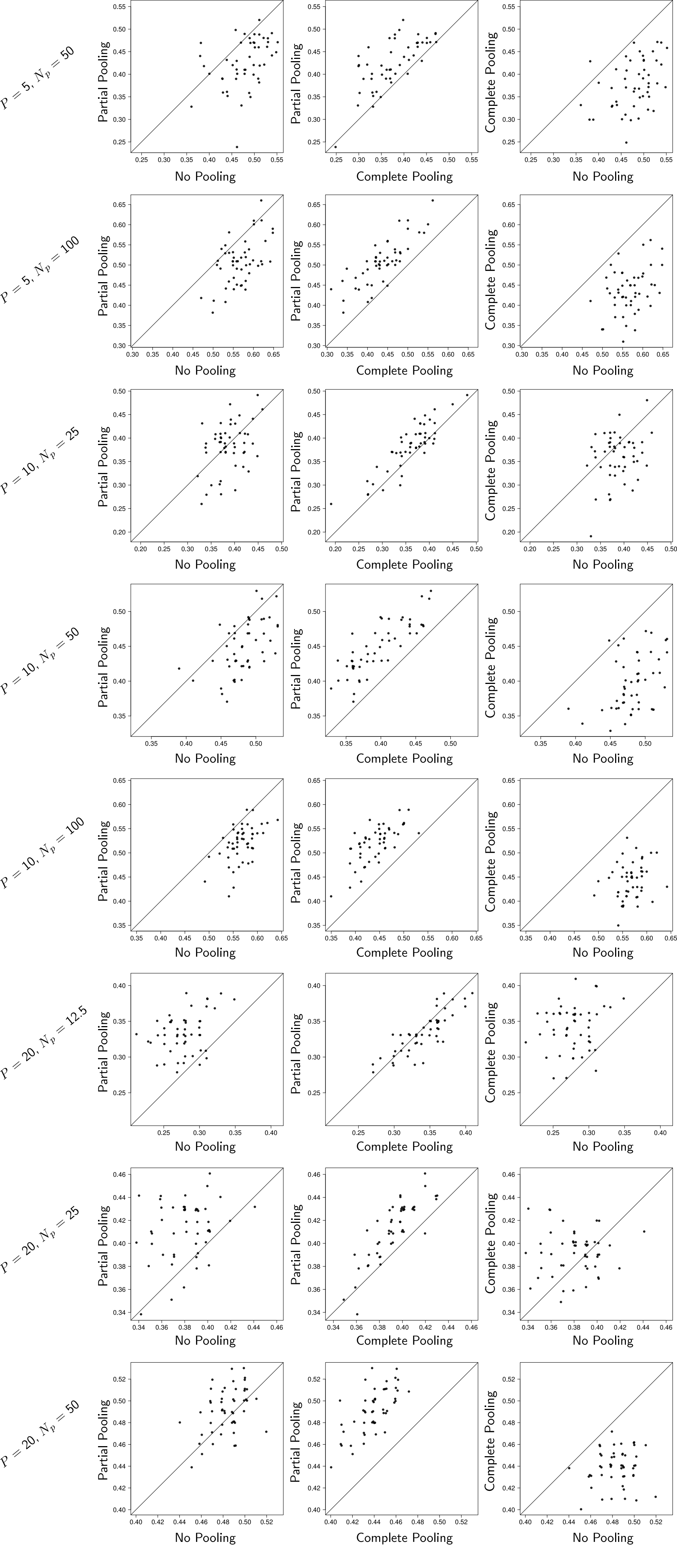

Figure S7 Prediction accuracy r_{Π} (for populations represented in training set) for trait ear height in the NAM population. The points correspond to the replications of the cross-validation. The number of populations in the training set is P and the number of individuals per population is N_p. The number of markers used was 575.

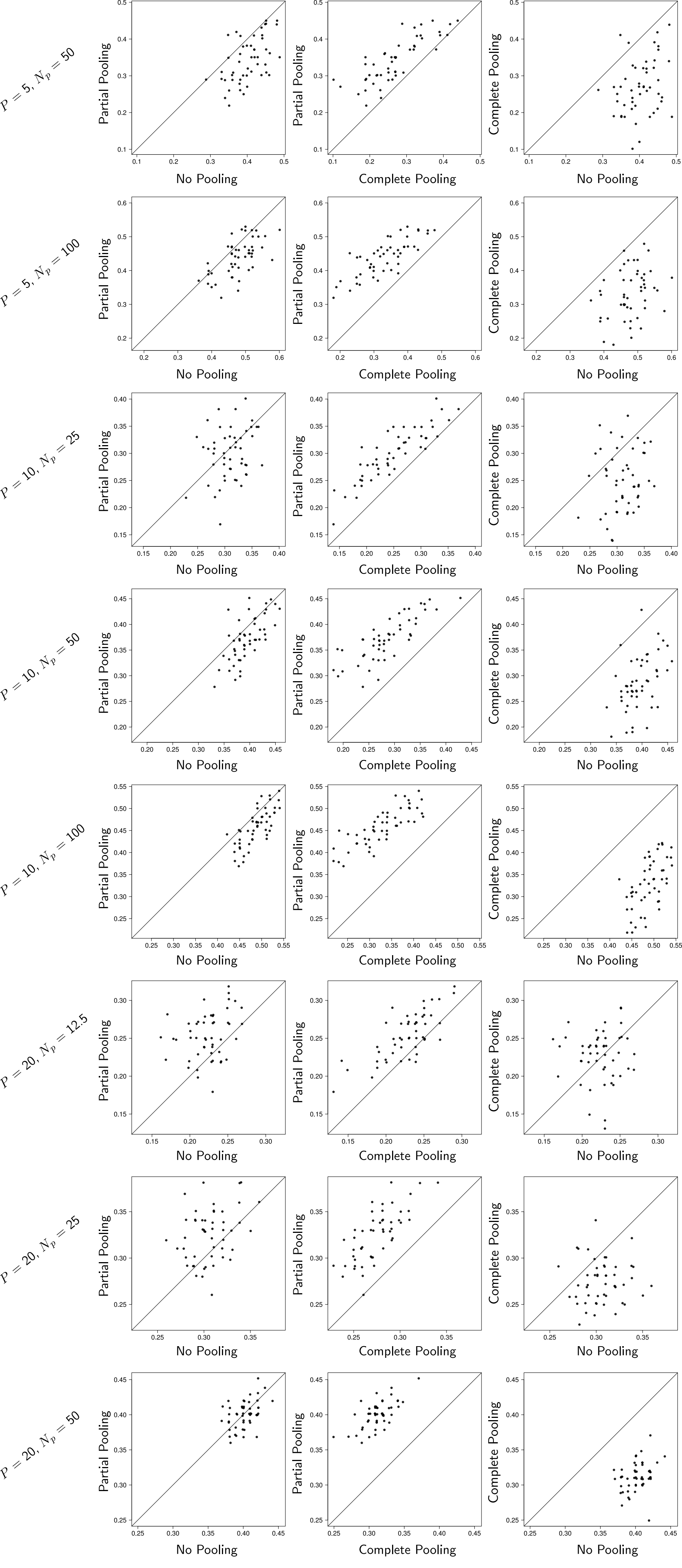

Figure S8 Prediction accuracy r_{Π} (for populations represented in training set) for trait ear length in the NAM population. The points correspond to the replications of the cross-validation. The number of populations in the training set is P and the number of individuals per population is N_p. The number of markers used was 575.

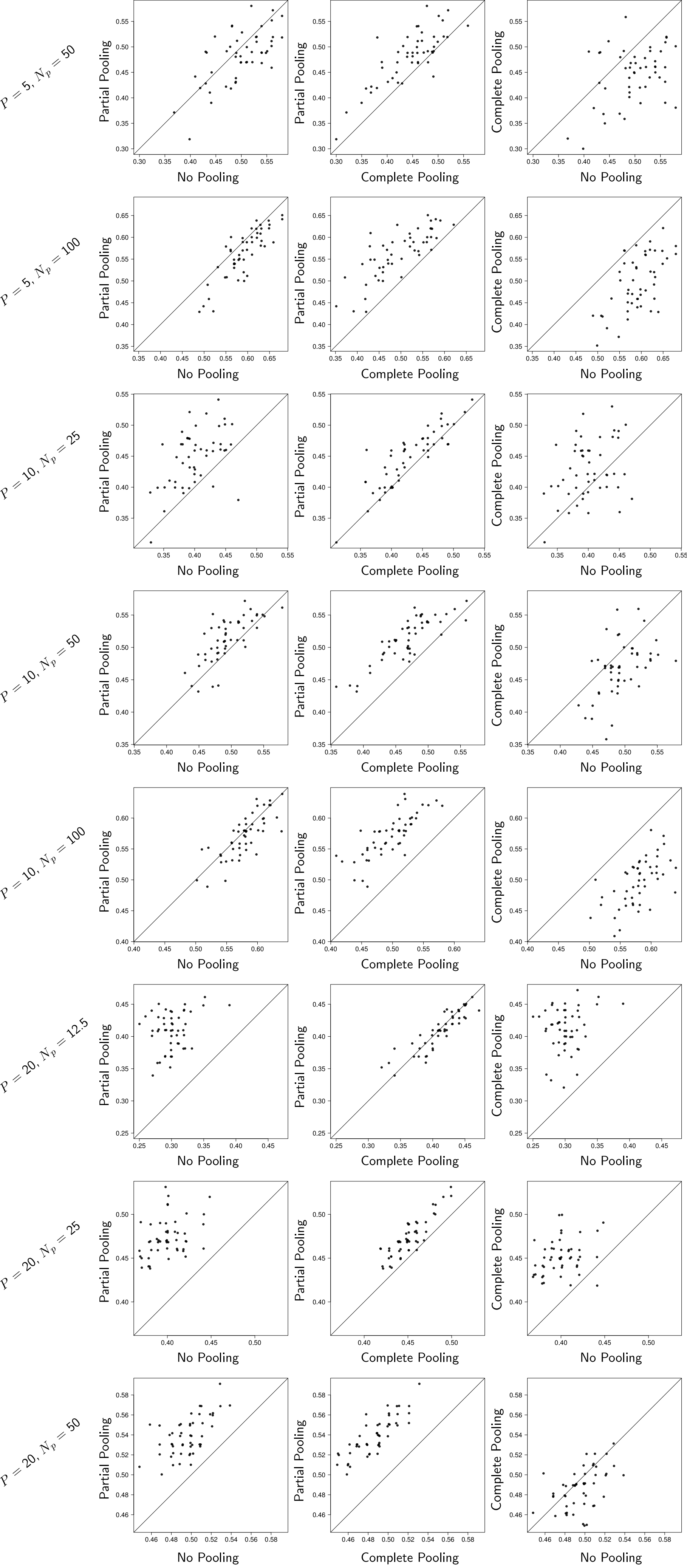

Figure S9 Prediction accuracy r_{II} (for populations represented in training set) for trait southern leaf blight in the NAM population. The points correspond to the replications of the cross-validation. The number of populations in the training set is P and the number of individuals per population is N_p. The number of markers used was 575.

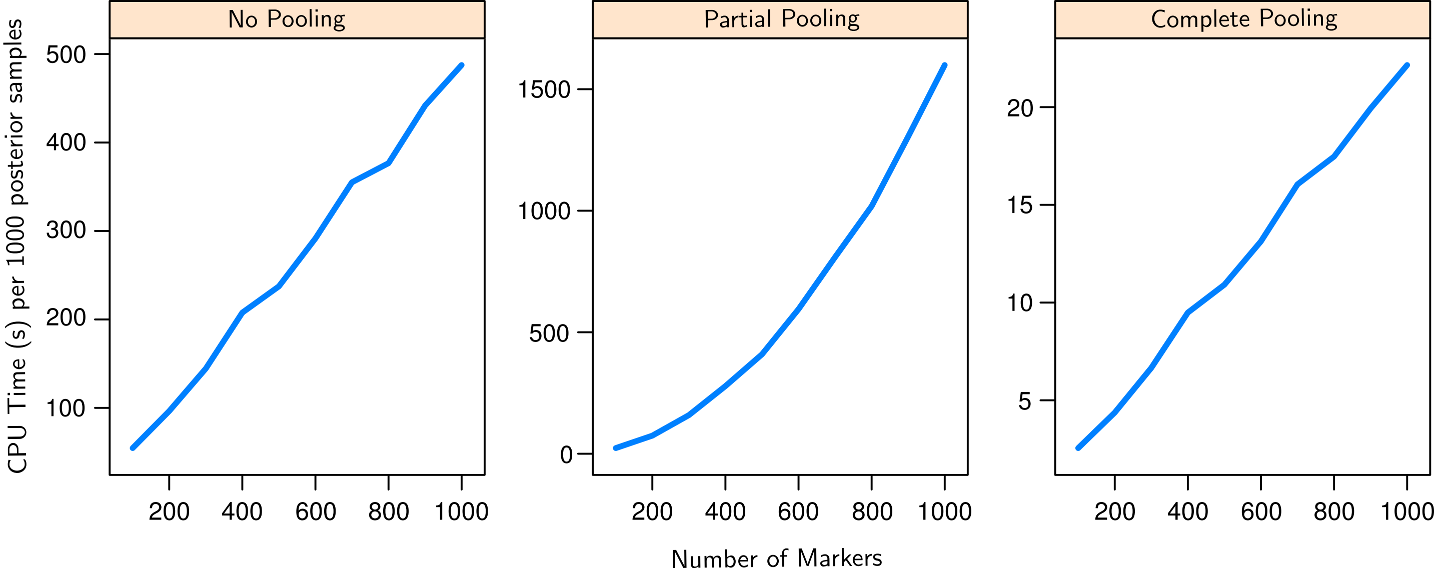

Figure S10 Average CPU time in seconds per 1000 posterior samples for pooling approaches with increasing number of markers. The trait was southern leaf blight, the number of populations was 20 and the number of indiviudals per population 25.

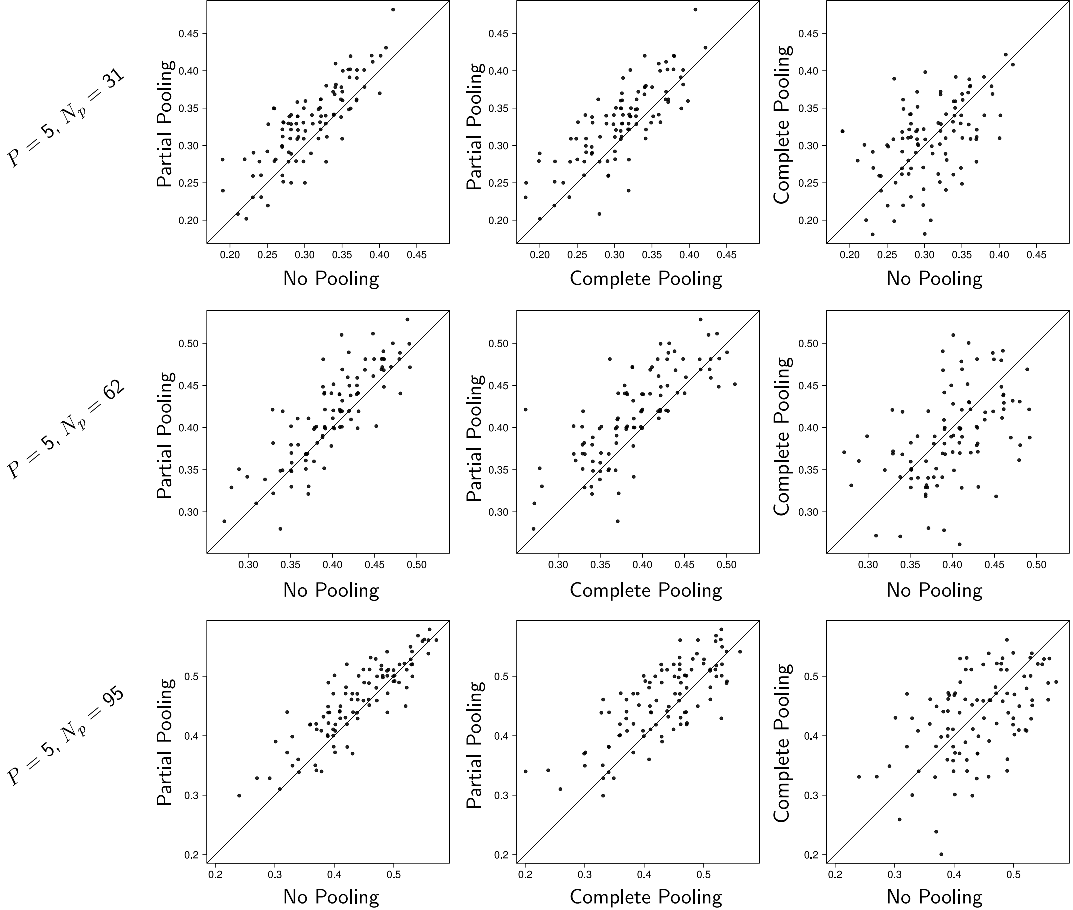

Figure S11 Prediction accuracy r_{Π} (for populations represented in training set) for trait ear length in the interconnected biparental maize population. The points correspond to the replications of the cross-validation. The number of populations in the training set is P and the average number of individuals per population is N_p. The number of markers used was 285.

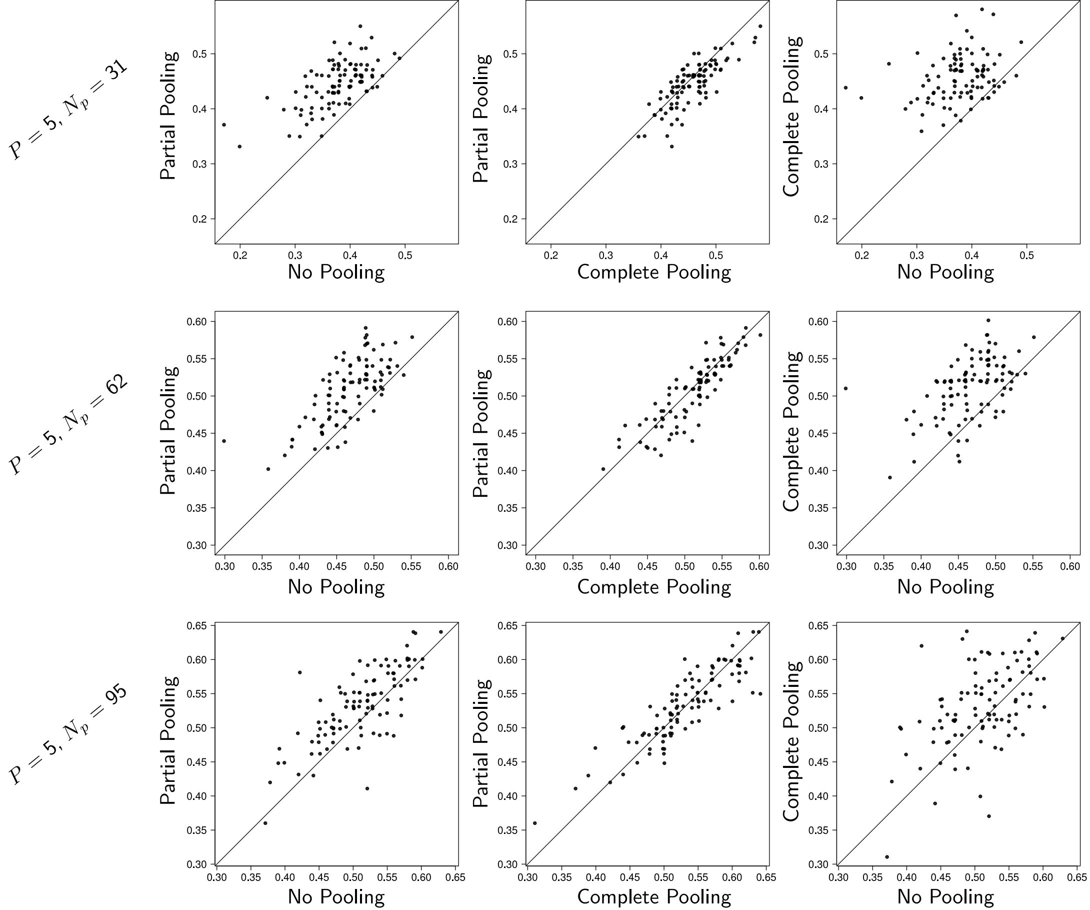

Figure S12 Prediction accuracy r_{Π} (for populations represented in training set) for trait deoxinivalenol content in the interconnected biparental maize population. The points correspond to the replications of the cross-validation. The number of populations in the training set is P and the average number of individuals per population is N_p. The number of markers used was 285.

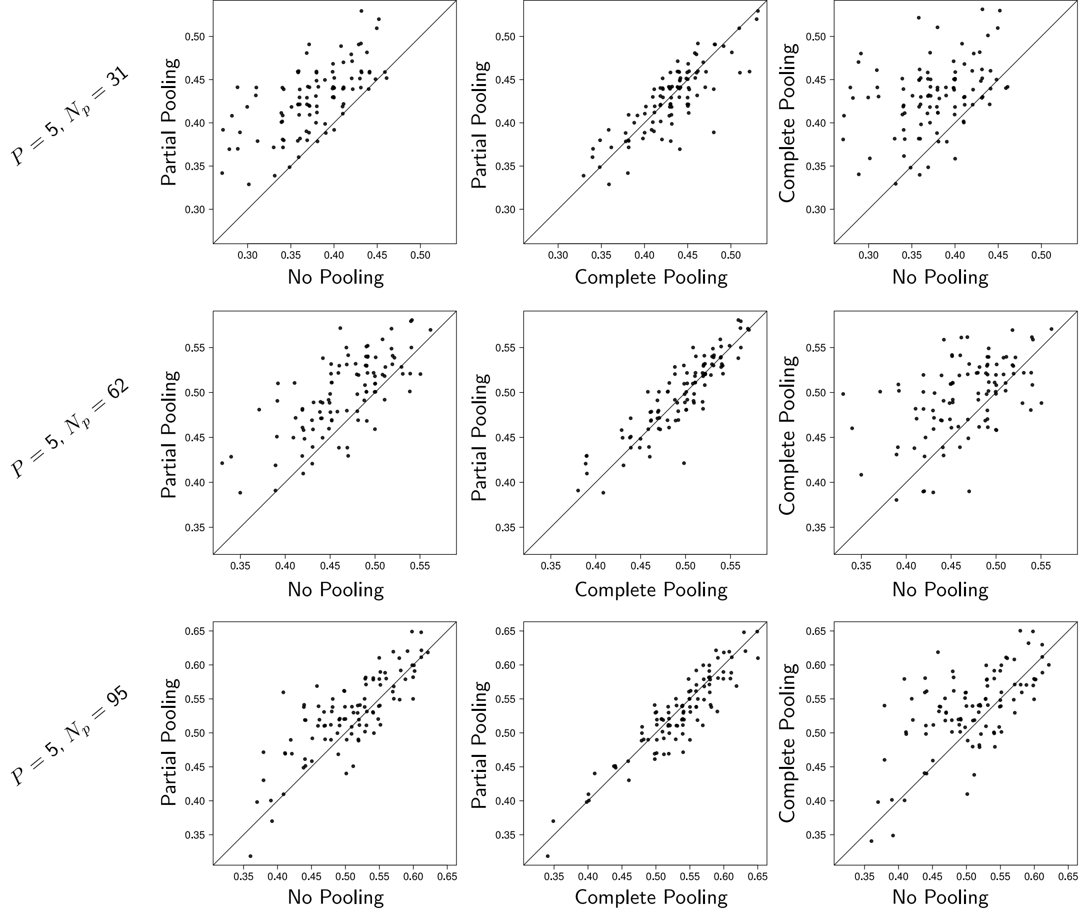

Figure S13 Prediction accuracy r_Π (for populations represented in training set) for trait Giberella ear rot severity in the interconnected biparental maize populations. The points correspond to the replications of the cross-validation. The number of populations in the training set is P and the average number of individuals per population is N_p. The number of markers used was 285.

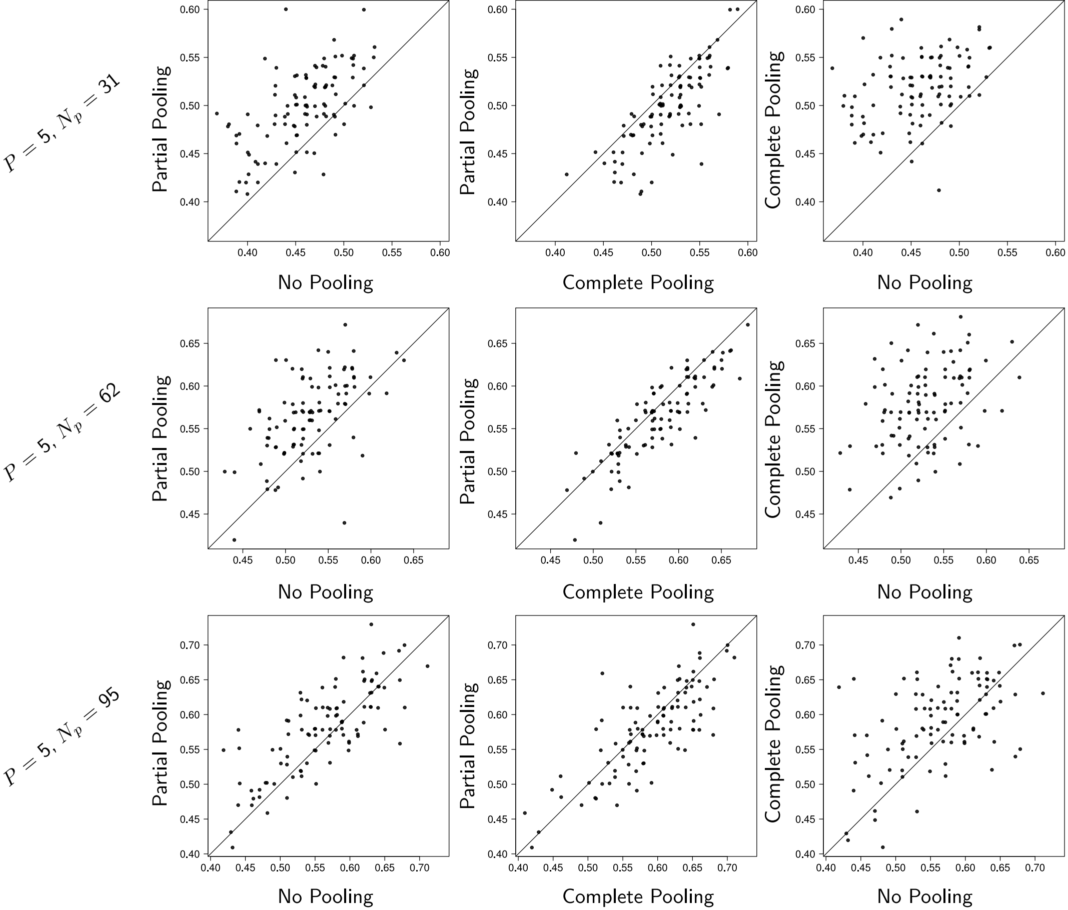

Figure S14 Prediction accuracy r_{Π} (for populations represented in training set) for trait kernel rows in the interconnected biparental maize populations. The points correspond to the replications of the cross-validation. The number of populations in the training set is P and the average number of individuals per population is N_p. The number of markers used was 285.

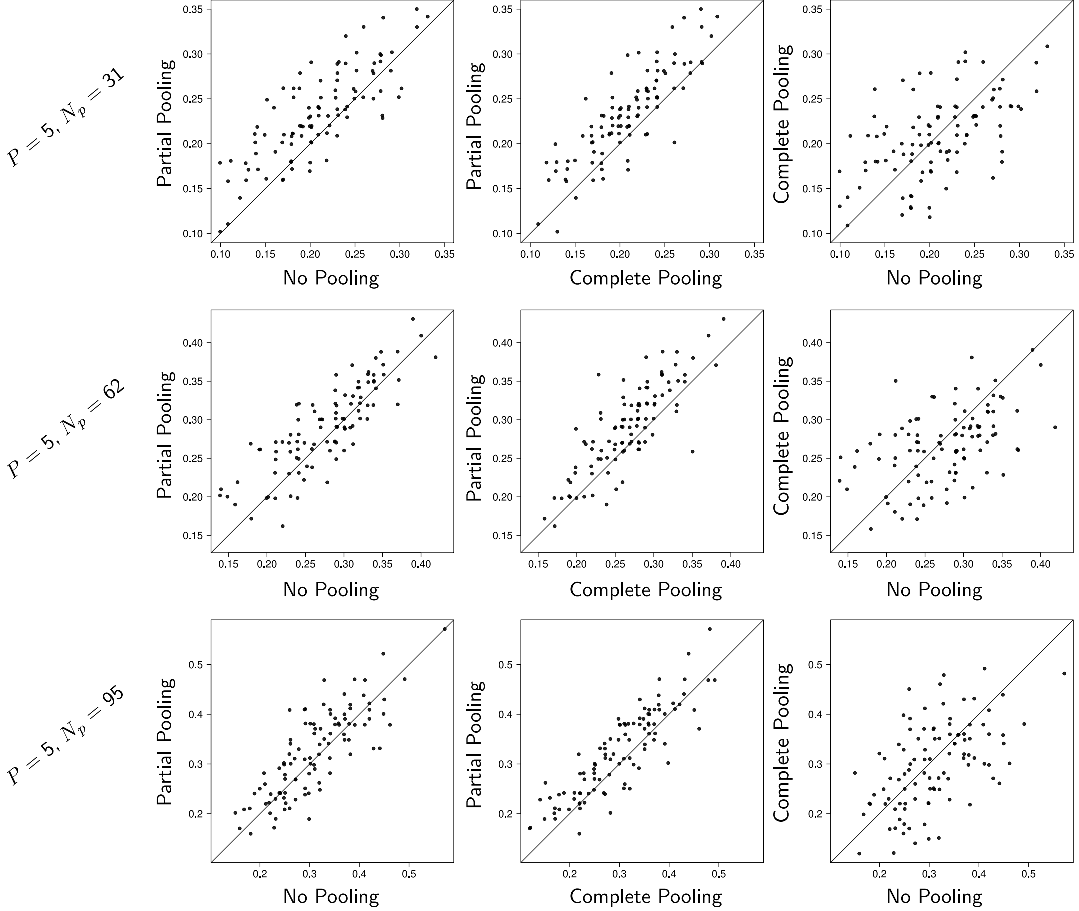

Figure S15 Prediction accuracy r_{Π} (for populations represented in training set) for trait kernels per row in the interconnected biparental maize populations. The points correspond to the replications of the cross-validation. The number of populations in the training set is P and the average number of individuals per population is N_p. The number of markers used was 285.

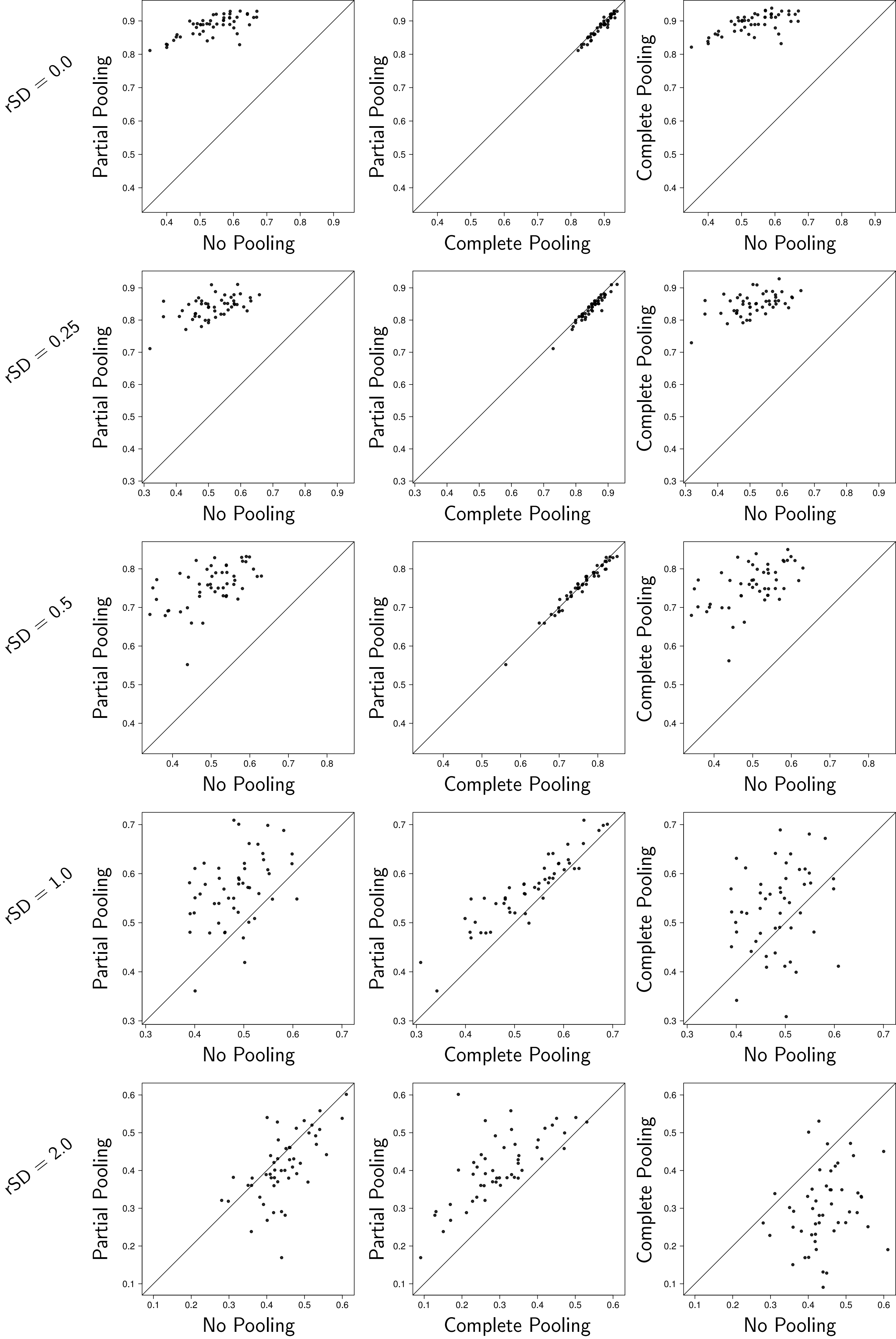

Figure S16 Prediction accuracy r_{Π} (for populations represented in training set) in simulated maize populations. The points correspond to the replications of the cross-validation. rSD is the relative standard deviation of simulated population specific QTL effects. The number of populations represented in the training set was 10 and the number of individuals per population 25. The number of markers used was 285.
