# Supplementary material for: Using Bayesian Multilevel Whole Genome Regression Models for Partial Pooling of Training Sets in Genomic Prediction
Source: G3 (Bethesda). 2015 May 29;5(8):1603–12. doi: 10.1534/g3.115.019299 (PMC4528317; doi:10.1534/g3.115.019299)
Supplement: Supporting Information [file supp_g3.115.019299_TableS1.pdf]

Table S1: Average within population prediction accuracies in NAM maize populations with 285 markers

			r_{Π}			$r_{\overline{\Pi}}$	
P	N_p	trait	no pooling	partial pooling	complete pooling	partial pooling	complete pooling
5	50	DS	0.41^{a}	0.34^{b}	0.2^{c}	0.19^{a}	0.19^{a}
		EH	0.47^{a}	0.44^{b}	0.39^{c}	0.31^{a}	0.32^{b}
		EL	0.39^{a}	0.37^{b}	0.28^{c}	0.19^{a}	0.19^{b}
		NS	0.39^{a}	0.37^{b}	0.32^{c}	0.25^{a}	0.26^{b}
		SLB	0.49^{a}	0.49^{a}	0.45^{b}	0.37^{a}	0.37^{b}
		ULA	0.50^{a}	0.48^{b}	0.44^{c}	0.36^{a}	0.36^{a}
	100	DS	0.52^{a}	0.41^{b}	0.28^{c}	0.21^{a}	0.20^{b}
		EH	0.57^{a}	0.51^{b}	0.43^{c}	0.34^{a}	0.34^{a}
		EL	0.49^{a}	0.46^{b}	0.35^{c}	0.23^{a}	0.23^{b}
		NS	0.47^{a}	0.44^{b}	0.36^{c}	0.29^{a}	0.29^{a}
		SLB	0.58^{a}	0.58^{a}	0.50^{b}	0.41^{a}	0.41^{a}
		ULA	0.58^{a}	0.54^{b}	0.47^{c}	0.40^{a}	0.40^{a}
10	25	DS	0.32^{a}	0.28^{b}	0.22^{c}	0.18^{a}	0.17^{b}
		EH	0.38^{a}	0.38^{a}	0.35^{b}	0.30^{a}	0.31^{b}
		EL	0.31^{a}	0.31^{a}	0.25^{b}	0.21^{a}	0.21^{a}
		NS	0.30^{a}	0.33^{b}	0.30^{a}	0.26^{a}	0.27^{b}
		SLB	0.40^{a}	0.46^{b}	0.43^{c}	0.38^{a}	0.39^{b}
		ULA	0.39^{a}	0.44^{b}	0.41^{c}	0.36^{a}	0.37^{a}
	50	DS	0.42^{a}	0.35^{b}	0.26^{c}	0.22^{a}	0.22^{a}
		EH	0.47^{a}	0.45^{b}	0.40^{c}	0.36^{a}	0.36^{b}
		EL	0.40^{a}	0.39^{b}	0.29^{c}	0.23^{a}	0.23^{a}
		NS	0.38^{a}	0.40^{b}	0.35^{c}	0.30^{a}	0.30^{b}
		SLB	0.49^{a}	0.52^{b}	0.46^{c}	0.42^{a}	0.43^{b}
		ULA	0.48	0.50	0.45	0.41^{a}	0.41^{a}
	100	DS	0.51^{a}	0.42^{b}	0.30^{c}	0.25^{a}	0.25^{b}
		EH	0.57^{a}	0.53^{b}	0.44^{c}	0.39^{a}	0.39^{b}
		EL	0.48^{a}	0.46^{b}	0.33^{c}	0.27^{a}	0.27^{a}
		NS	0.48^{a}	0.46^{b}	0.38^{c}	0.33^{a}	0.33^{a}
		SLB	0.57^{a}	0.57^{a}	0.49^{b}	0.45^{a}	0.45^{b}
		ULA	0.59^{a}	0.56^{b}	0.48^{c}	0.45^{a}	0.44^{b}
20	12.5	DS	0.23^{a}	0.23^{a}	0.21^{b}	0.17^{a}	0.17^{a}
		EH	0.28^{a}	0.34^{b}	0.33^{c}	0.30^{a}	0.31^{b}
		EL	0.22^{a}	0.27^{b}	0.23^{a}	0.19^{a}	0.19^{a}
		NS	0.21^{a}	0.30^{b}	0.29^{c}	0.27^{a}	0.28^{a}
		SLB	0.31^{a}	0.43^{b}	0.42^{c}	0.38^{a}	0.39^{a}
		ULA	0.28^{a}	0.40^{b}	0.39^{c}	0.35^{a}	0.36^{a}
	25	DS	0.32^{a}	0.30^{b}	0.24^{c}	0.22^{a}	0.23^{a}
		EH	0.38^{a}	0.42^{b}	0.39^{c}	0.36^{a}	0.37^{b}

Continued on next page

	EL	0.31[a]	0.34[b]	0.28[c]	0.22[a]	0.22[a]
	NS	0.30[a]	0.36[b]	0.33[b]	0.30[a]	0.31[b]
	SLB	0.39[a]	0.48[b]	0.45[c]	0.42[a]	0.43[b]
	ULA	0.38[a]	0.46[b]	0.44[c]	0.42[a]	0.42[a]
50	DS	0.42[a]	0.37[b]	0.29[c]	0.26[a]	0.26[a]
	EH	0.48[a]	0.49[b]	0.42[c]	0.40[a]	0.40[a]
	EL	0.39[a]	0.40[a]	0.30[b]	0.28[a]	0.29[b]
	NS	0.38[a]	0.41[b]	0.36[c]	0.34[a]	0.34[a]
	SLB	0.49[a]	0.54[b]	0.48[c]	0.46[a]	0.47[b]
	ULA	0.49[a]	0.52[b]	0.47[c]	0.46[a]	0.46[a]

Values shown are average within population prediction accuracies for test individuals, averaged over 50 random estimation-test data splits. Values with common letters within a row and within either r_{Π} or $r_{\overline{\Pi}}$ are not significantly different in paired t-tests at an alpha level of 0.05. The standard errors of the averages were $<$ 0.013. r_{Π} is the prediction accuracy for populations represented in the training set and $r_{\overline{\Pi}}$ the prediction accuracy of populations not represented in the training set. P gives the size of set Π, i.e., the number of populations represented in the training set, column N_p gives the number of individuals from each population in Π that were used for estimation, i.e., the sizes of sets Λ_p. The traits are: days to silking (DS), ear height (EH), ear length (EL), southern leaf blight resistance (SLB), near-infrared starch measurements (NS) and upper leaf angle (ULA).
