# Supplementary material for: Using Bayesian Multilevel Whole Genome Regression Models for Partial Pooling of Training Sets in Genomic Prediction
Source: G3 (Bethesda). 2015 May 29;5(8):1603–12. doi: 10.1534/g3.115.019299 (PMC4528317; doi:10.1534/g3.115.019299)
Supplement: Supporting Information [file supp_g3.115.019299_TableS2.pdf]

TABLE S2: Anova for the influence of factors on prediction accuracy of populations represented in the training set (r_{Π}) for the NAM populations with 285 markers

Source	Df	Sum Sq	Mean Sq	F value	Pr(>F)
pooling	2	5.23	2.62	3223.38	0.0000
trait	5	24.06	4.81	5931.88	0.0000
N_p	1	22.58	22.58	27824.73	0.0000
P	1	0.07	0.07	86.06	0.0000
replication	2391	10.90	0.00	5.62	0.0000
pooling:trait	10	2.52	0.25	310.73	0.0000
pooling:N_p	2	3.92	1.96	2417.16	0.0000
pooling:P	2	0.38	0.19	233.29	0.0000
pooling:trait:N_p	10	0.08	0.01	10.08	0.0000
pooling:trait:P	10	0.02	0.00	2.15	0.0178
pooling:N_p:P	2	0.23	0.11	141.71	0.0000
pooling:trait:N_p:P	10	0.02	0.00	1.95	0.0345
Residuals	4750	3.85	0.00		

Degrees of freedom (Df), sum of squares (Sum Sq), mean squares (Mean Sq). The pooling approaches are referred to as 'pooling'
