# Supplementary material for: Using Bayesian Multilevel Whole Genome Regression Models for Partial Pooling of Training Sets in Genomic Prediction
Source: G3 (Bethesda). 2015 May 29;5(8):1603–12. doi: 10.1534/g3.115.019299 (PMC4528317; doi:10.1534/g3.115.019299)
Supplement: Supporting Information [file supp_g3.115.019299_TableS3.pdf]

TABLE S3: Average within population prediction accuracies in NAM maize populations using 575 markers

			r_{Π}			$r_{\overline{\Pi}}$	
P	N_p	trait	no pooling	partial pooling	complete pooling	partial pooling	complete pooling
5	50	EH	0.48^{a}	0.43^{b}	0.38^{c}	0.29^{a}	0.31^{b}
		EL	0.40^{a}	0.34^{b}	0.27^{c}	0.19^{a}	0.19^{a}
		SLB	0.50^{a}	0.48^{b}	0.45^{c}	0.36^{a}	0.37^{b}
	100	EH	0.57^{a}	0.51^{b}	0.44^{c}	0.34^{a}	0.35^{b}
		EL	0.48^{a}	0.44^{b}	0.33^{c}	0.21^{a}	0.21^{a}
		SLB	0.59^{a}	0.56^{b}	0.50^{c}	0.40^{a}	0.41^{b}
10	25	EH	0.39^{a}	0.38^{a}	0.36^{b}	0.30^{a}	0.32^{b}
		EL	0.31^{a}	0.30^{a}	0.25^{b}	0.20^{a}	0.20^{b}
		SLB	0.40^{a}	0.45^{b}	0.43^{c}	0.37^{a}	0.39^{b}
	50	EH	0.48^{a}	0.45^{b}	0.40^{c}	0.35^{a}	0.36^{b}
		EL	0.39^{a}	0.37^{b}	0.29^{c}	0.24^{a}	0.24^{b}
		SLB	0.49^{a}	0.51^{b}	0.47^{c}	0.41^{a}	0.42^{b}
	100	EH	0.57^{a}	0.52^{b}	0.44^{c}	0.39^{a}	0.40^{b}
		EL	0.49^{a}	0.46^{b}	0.33^{c}	0.26^{a}	0.27^{b}
		SLB	0.58^{a}	0.57^{b}	0.50^{c}	0.44^{a}	0.45^{b}
20	12.5	EH	0.28^{a}	0.33^{b}	0.34^{c}	0.28^{a}	0.31^{b}
		EL	0.22^{a}	0.25^{b}	0.23^{a}	0.20^{a}	0.21^{b}
		SLB	0.30^{a}	0.41^{b}	0.41^{b}	0.37^{a}	0.39^{b}
	25	EH	0.38^{a}	0.41^{b}	0.39^{c}	0.37^{a}	0.39^{b}
		EL	0.31^{a}	0.32^{b}	0.27^{c}	0.25^{a}	0.26^{b}
		SLB	0.40^{a}	0.47^{b}	0.45^{c}	0.42^{a}	0.44^{b}
	50	EH	0.48^{a}	0.49^{b}	0.44^{c}	0.39^{a}	0.40^{b}
		EL	0.40^{a}	0.40^{a}	0.31^{b}	0.27^{a}	0.28^{a}
		SLB	0.50^{a}	0.54^{b}	0.49^{c}	0.45^{a}	0.46^{b}

Values shown are average within population prediction accuracies for test individuals, averaged over 50 random estimation-test data splits. Values with common letters within a row and within either $r\Pi$ or $r_{\overline{\Pi}}$ are not significantly different in paired t-tests at an alpha level of 0.05. The standard errors of the averages were $<$ 0.01. r_{Π} is the prediction accuracy for populations represented in the training set and $r_{\overline{\Pi}}$ the prediction accuracy of populations not represented in the training set. P gives the size of set Π, i.e., the number of populations represented in the training set, column N_p gives the number of individuals from each population in Π that were used for estimation, i.e., the sizes of sets Λ_p. The traits were: ear height (EH), ear length (EL) and southern leaf blight resistance (SLB).
