# Supplementary material for: Using Bayesian Multilevel Whole Genome Regression Models for Partial Pooling of Training Sets in Genomic Prediction
Source: G3 (Bethesda). 2015 May 29;5(8):1603–12. doi: 10.1534/g3.115.019299 (PMC4528317; doi:10.1534/g3.115.019299)
Supplement: Supporting Information [file supp_g3.115.019299_TableS4.pdf]

TABLE S4: Anova for the influence of factors on prediction accuracy of populations represented in the training set (r_{Π}) for the NAM populations with 575 markers

Source	Df	Sum Sq	Mean Sq	F value	Pr(>F)
pooling	2	2.19	1.10	1541.96	0.0000
trait	2	11.49	5.75	8084.31	0.0000
N_p	1	11.38	11.38	16016.89	0.0000
P	1	0.09	0.09	126.11	0.0000
replication	1195	5.46	0.00	6.43	0.0000
pooling:trait	4	0.72	0.18	254.16	0.0000
pooling:N_p	2	1.82	0.91	1283.11	0.0000
pooling:P	2	0.30	0.15	207.80	0.0000
pooling:trait:N_p	4	0.04	0.01	12.82	0.0000
pooling:trait:P	4	0.01	0.00	2.15	0.0718
pooling:N_p:P	2	0.13	0.07	94.45	0.0000
pooling:trait:N_p:P	4	0.00	0.00	0.33	0.8561
Residuals	2376	1.69	0.00		

Degrees of freedom (Df), sum of squares (Sum Sq), mean squares (Mean Sq). The pooling approaches are referred to as “pooling”
