# Supplementary material for: Using Bayesian Multilevel Whole Genome Regression Models for Partial Pooling of Training Sets in Genomic Prediction
Source: G3 (Bethesda). 2015 May 29;5(8):1603–12. doi: 10.1534/g3.115.019299 (PMC4528317; doi:10.1534/g3.115.019299)
Supplement: Supporting Information [file supp_g3.115.019299_TableS5.pdf]

TABLE S5: Average within population prediction accuracies in interconnected biparental maize populations

N_p	Trait	Pooling		
		no	partial	complete
31	EL	0.31[a]	0.33[b]	0.31[a]
	DON	0.38[a]	0.44[b]	0.46[c]
	GER	0.38[a]	0.43[b]	0.43[b]
	KR	0.46[a]	0.50[b]	0.52[c]
	KpR	0.21[a]	0.23[b]	0.21[a]
62	EL	0.40[a]	0.40[b]	0.39[a]
	DON	0.47[a]	0.51[b]	0.51[c]
	GER	0.47[a]	0.50[b]	0.49[b]
	KR	0.53[a]	0.56[b]	0.58[c]
	KpR	0.28[a]	0.29[b]	0.27[a]
95	EL	0.44[a]	0.46[b]	0.43[a]
	DON	0.51[a]	0.53[b]	0.53[b]
	GER	0.51[a]	0.53[b]	0.53[c]
	KR	0.56[a]	0.58[b]	0.59[c]
	KpR	0.31[a]	0.32[b]	0.30[a]

Values shown are average within population prediction accuracies for test individuals, averaged over 100 random estimation-test data splits. Values within a row with common letters are not significantly different at an alpha level of 0.05 in a paired t-test. Standard errors of the averages were $<$ 0.01. N_p denotes the average number of individuals per population in the training set. The traits were ear length (EL), deoxynivalenol content (DON), Giberella ear rot severity (GER) kernel rows (KR) and kernels per row (KpR)
