# Supplementary material for: Using Bayesian Multilevel Whole Genome Regression Models for Partial Pooling of Training Sets in Genomic Prediction
Source: G3 (Bethesda). 2015 May 29;5(8):1603–12. doi: 10.1534/g3.115.019299 (PMC4528317; doi:10.1534/g3.115.019299)
Supplement: Supporting Information [file supp_g3.115.019299_TableS6.pdf]

TABLE S6: Anova for the influence of factors on prediction accuracy of populations represented in the training set (r_{Π}) for the interconnected biparental maize populations

Source	Df	Sum Sq	Mean Sq	F value	Pr(>F)
pooling	2	0.72	0.36	397.65	0.0000
trait	4	40.67	10.17	11260.24	0.0000
N_p	1	8.01	8.01	8866.52	0.0000
replication	1494	10.99	0.01	8.14	0.0000
pooling:trait	8	0.48	0.06	66.38	0.0000
pooling:N_p	2	0.11	0.05	59.21	0.0000
pooling:trait:N_p	8	0.05	0.01	6.56	0.0000
Residuals	2980	2.69	0.00		

Degrees of freedom (Df), sum of squares (Sum Sq), mean squares (Mean Sq). The pooling approaches are referred to as “pooling”
