# Supplementary material for: Using Bayesian Multilevel Whole Genome Regression Models for Partial Pooling of Training Sets in Genomic Prediction
Source: G3 (Bethesda). 2015 May 29;5(8):1603–12. doi: 10.1534/g3.115.019299 (PMC4528317; doi:10.1534/g3.115.019299)
Supplement: Supporting Information [file supp_g3.115.019299_TableS7.pdf]

TABLE S7: Average prediction accuracies for simulated maize populations

	r_{Π}			$r_{\overline{\Pi}}$	
rSD	no pooling	partial pooling	complete pooling	partial pooling	complete pooling
0.0	0.54[a]	0.89[b]	0.89[c]	0.89[a]	0.89[b]
0.25	0.51[a]	0.84[b]	0.85[c]	0.84[a]	0.84[b]
0.5	0.50[a]	0.76[b]	0.76[b]	0.73[a]	0.73[b]
1.0	0.48[a]	0.57[b]	0.53[c]	0.48[a]	0.49[b]
2.0	0.44[a]	0.41[b]	0.30[c]	0.20[a]	0.21[b]

Values shown are average within population prediction accuracies for test individuals, averaged over 50 random estimation-test data splits. Standard errors were < 0.015. r_{Π} is the prediction accuracy for populations represented in the training set and $r_{\overline{\Pi}}$ the prediction accuracy of populations not represented in the training set. rSD is the relative standard deviation of simulated population specific QTL effects.
