# Supplementary material for: Using Bayesian Multilevel Whole Genome Regression Models for Partial Pooling of Training Sets in Genomic Prediction
Source: G3 (Bethesda). 2015 May 29;5(8):1603–12. doi: 10.1534/g3.115.019299 (PMC4528317; doi:10.1534/g3.115.019299)
Supplement: Supporting Information [file supp_g3.115.019299_TableS8.pdf]

TABLE S8: Anova for the influence of factors on prediction accuracy of populations represented in the training set (r_{Π}) for the simulated maize populations

Source	Df	Sum Sq	Mean Sq	F value	Pr(>F)
pooling	2	7.14	3.57	1786.49	0.0000
rSD	5	17.04	3.41	1706.58	0.0000
replication	269	2.36	0.01	4.39	0.0000
pooling:rSD	10	5.41	0.54	270.67	0.0000
Residuals	538	1.07	0.00		

Degrees of freedom (Df), sum of squares (Sum Sq), mean squares (Mean Sq). The pooling approaches are referred to as "pooling", rSD is the relative standard deviation of simulated population specific QTL effects
