# Supplementary figures and images for: Using Bayesian Multilevel Whole Genome Regression Models for Partial Pooling of Training Sets in Genomic Prediction
Source: G3 (Bethesda). 2015 May 29;5(8):1603–12. doi: 10.1534/g3.115.019299 (PMC4528317; doi:10.1534/g3.115.019299)

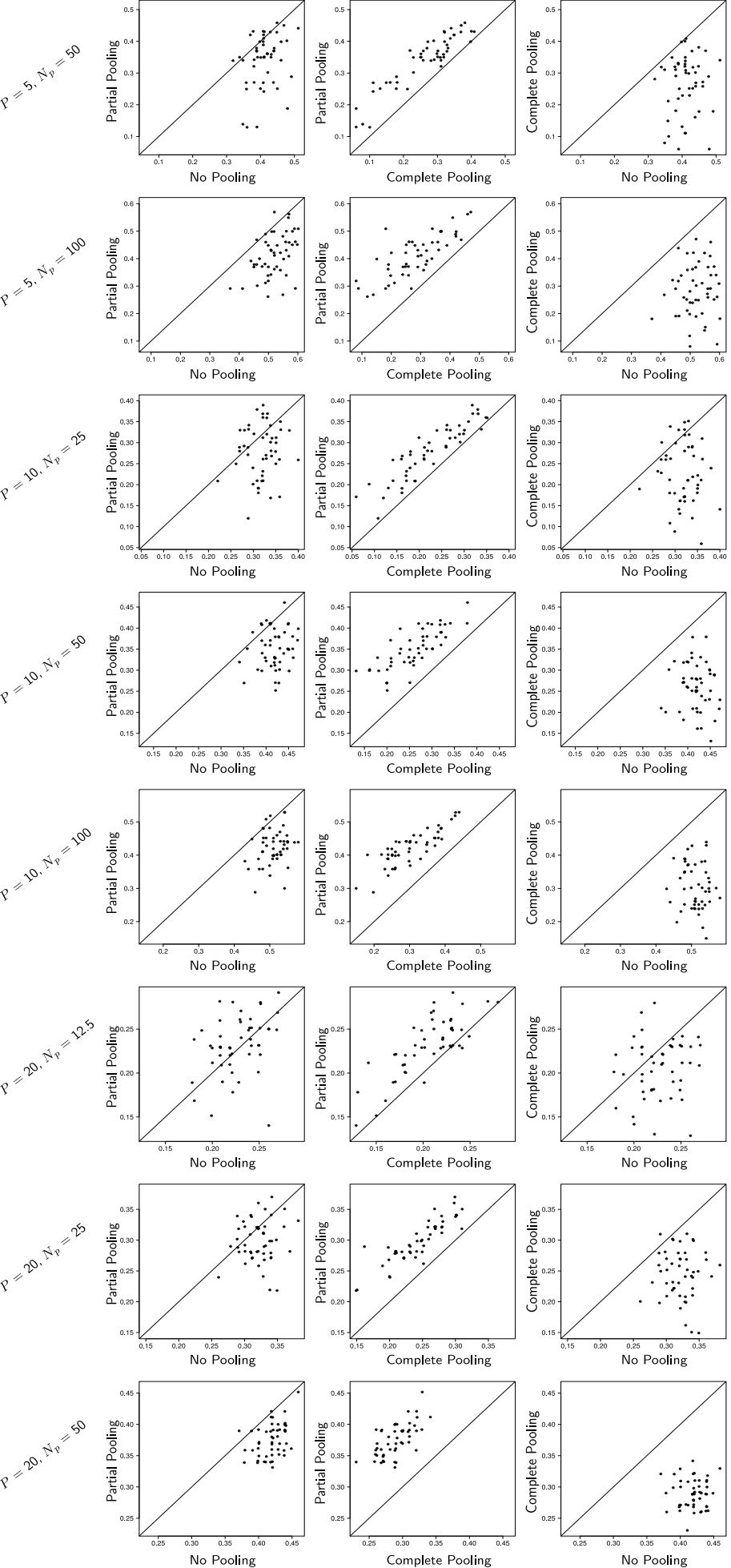

Supplement: Supporting Information [file supp_g3.115.019299_FigureS1.pdf]
