# Supplementary material for: Using Bayesian Multilevel Whole Genome Regression Models for Partial Pooling of Training Sets in Genomic Prediction
Source: G3 (Bethesda). 2015 May 29;5(8):1603–12. doi: 10.1534/g3.115.019299 (PMC4528317; doi:10.1534/g3.115.019299)
Supplement: Supporting Information [file supp_g3.115.019299_FigureS8.pdf]

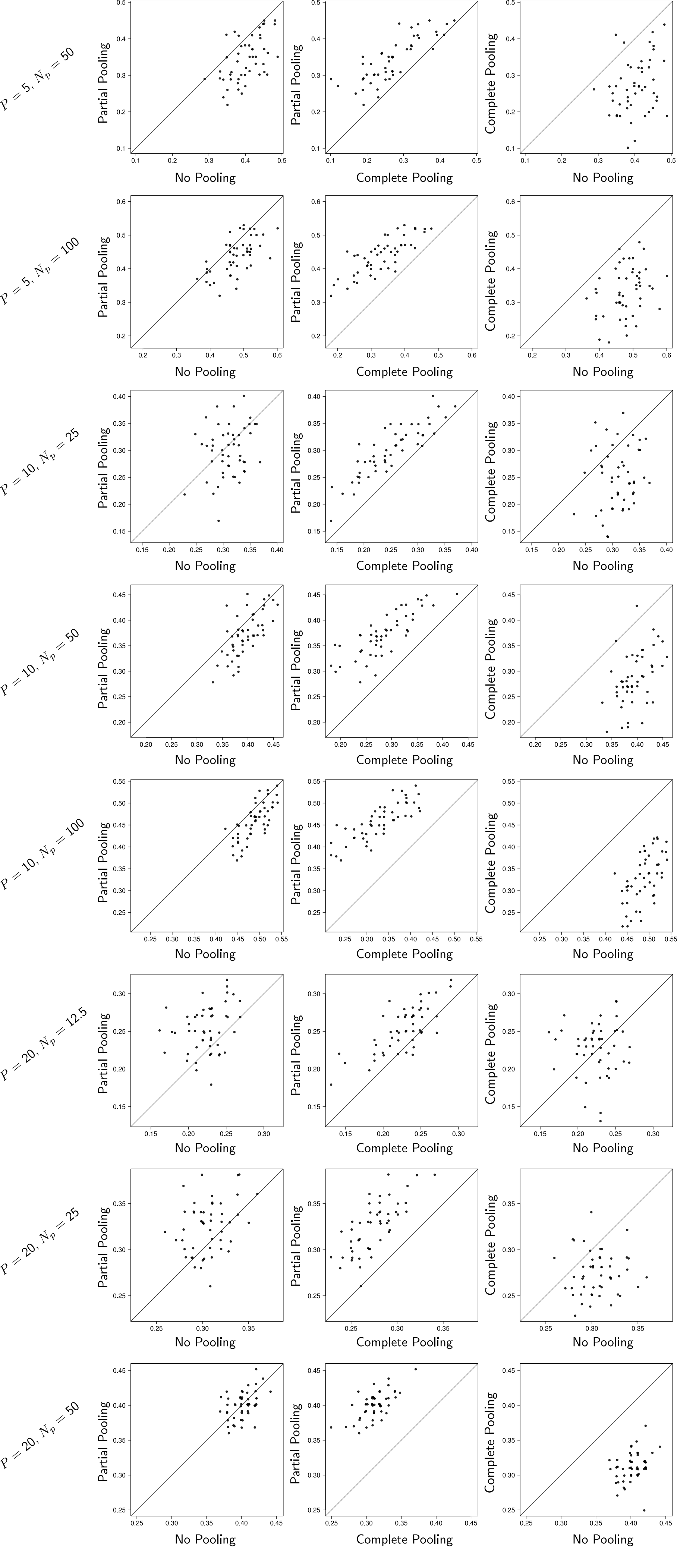

Figure S8 Prediction accuracy r_{Π} (for populations represented in training set) for trait ear length in the NAM population. The points correspond to the replications of the cross-validation. The number of populations in the training set is P and the number of individuals per population is N_p. The number of markers used was 575.
