# Supplementary material for: Using Bayesian Multilevel Whole Genome Regression Models for Partial Pooling of Training Sets in Genomic Prediction
Source: G3 (Bethesda). 2015 May 29;5(8):1603–12. doi: 10.1534/g3.115.019299 (PMC4528317; doi:10.1534/g3.115.019299)
Supplement: Supporting Information [file supp_g3.115.019299_FigureS10.pdf]

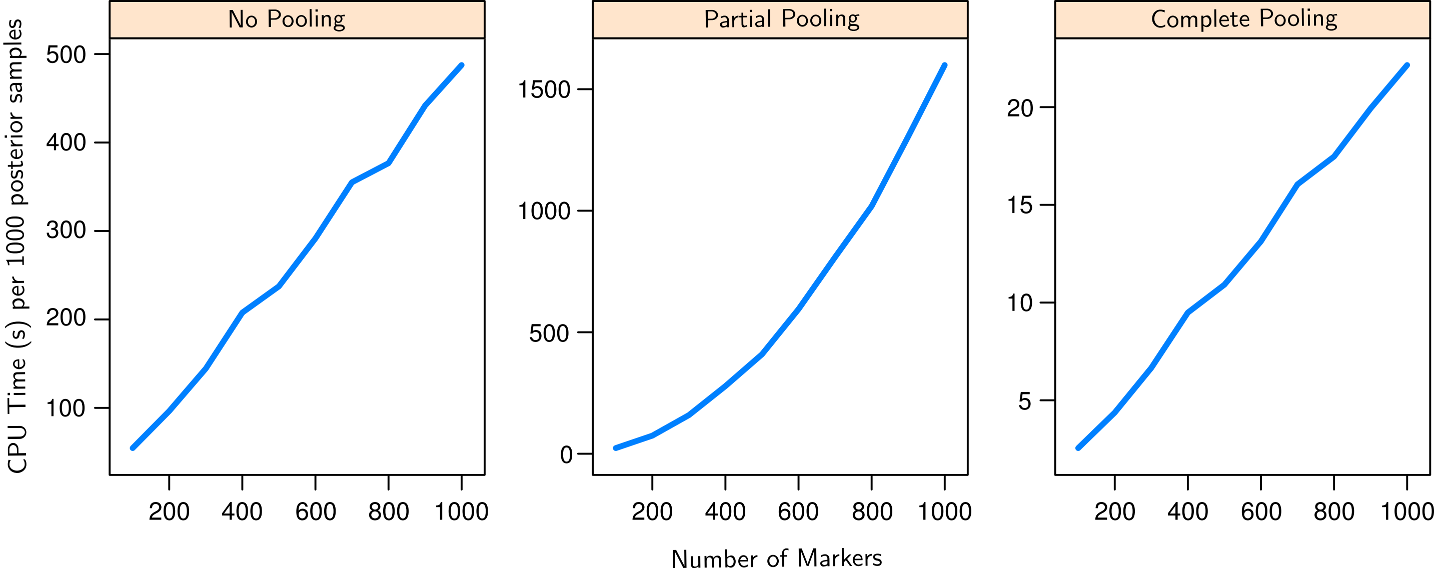

Figure S10 Average CPU time in seconds per 1000 posterior samples for pooling approaches with increasing number of markers. The trait was southern leaf blight, the number of populations was 20 and the number of indiviudals per population 25.
