# Supplementary material for: Using Bayesian Multilevel Whole Genome Regression Models for Partial Pooling of Training Sets in Genomic Prediction
Source: G3 (Bethesda). 2015 May 29;5(8):1603–12. doi: 10.1534/g3.115.019299 (PMC4528317; doi:10.1534/g3.115.019299)
Supplement: Supporting Information [file supp_g3.115.019299_FigureS16.pdf]

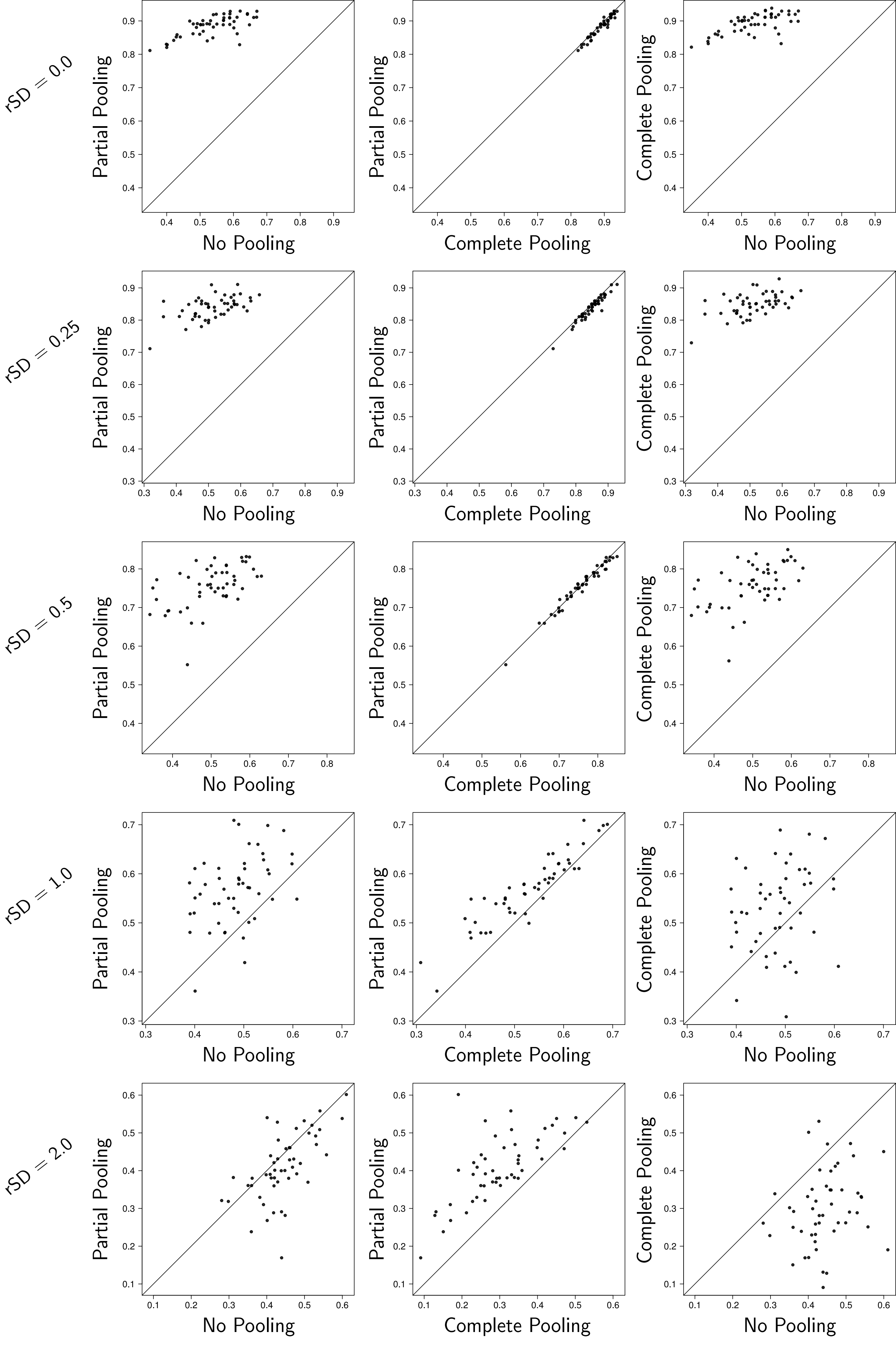

Figure S16 Prediction accuracy r_Π (for populations represented in training set) in simulated maize populations. The points correspond to the replications of the cross-validation. rSD is the relative standard deviation of simulated population specific QTL effects. The number of populations represented in the training set was 10 and the number of individuals per population 25. The number of markers used was 285.
